# Identification, prevalence and pathogenicity of *Colletotrichum* species causing anthracnose of *Capsicum annuum* in Asia

**DOI:** 10.1186/s43008-019-0001-y

**Published:** 2019-06-28

**Authors:** Dilani D. de Silva, Johannes Z. Groenewald, Pedro W. Crous, Peter K. Ades, Andi Nasruddin, Orarat Mongkolporn, Paul W. J. Taylor

**Affiliations:** 1 Faculty of Veterinary and Agricultural Sciences, The University of Melbourne, Parkville, VIC 3010 Australia; 2 Westerdijk Fungal Biodiversity Institute, Uppsalalaan 8, 3584 CT Utrecht, The Netherlands; 3 Faculty of Science, The University of Melbourne, Parkville, VIC 3010 Australia; 4 Department of Plant Pest & Disease, Universitas Hasanuddin, Makassar, Indonesia; 5 Department of Horticulture, Faculty of Agriculture at Kamphaeng Saen, Kasetsart University, Kamphaeng Saen Campus, Nakhon Pathom, Thailand

**Keywords:** Phylogenetics, Plant pathology, New taxa

## Abstract

Anthracnose of chili (*Capsicum* spp.) causes major production losses throughout Asia where chili plants are grown. A total of 260 *Colletotrichum* isolates, associated with necrotic lesions of chili leaves and fruit were collected from chili producing areas of Indonesia, Malaysia, Sri Lanka, Thailand and Taiwan. *Colletotrichum truncatum* was the most commonly isolated species from infected chili fruit and was readily identified by its falcate spores and abundant setae in the necrotic lesions. The other isolates consisted of straight conidia (cylindrical and fusiform) which were difficult to differentiate to species based on morphological characters. Taxonomic analysis of these straight conidia isolates based on multi-gene phylogenetic analyses (ITS*, gapdh, chs-1, act, tub2, his3, ApMat, gs*) revealed a further seven known *Colletotrichum* species, *C. endophyticum, C. fructicola, C. karsti, C. plurivorum, C. scovillei*, *C. siamense* and *C. tropicale*. In addition, three novel species are also described as *C. javanense, C. makassarense* and *C. tainanense*, associated with anthracnose of chili fruit in West Java (Indonesia); Makassar, South Sulawesi (Indonesia); and Tainan (Taiwan), respectively. *Colletotrichum siamense* is reported for the first time causing anthracnose of *Capsicum annuum* in Indonesia and Sri Lanka. This is also the first report of *C. fructicola* causing anthracnose of chili in Taiwan and Thailand and *C. plurivorum* in Malaysia and Thailand. Of the species with straight conidia, *C. scovillei* (acutatum complex), was the most prevalent throughout the surveyed countries, except for Sri Lanka from where this species was not isolated. *Colletotrichum siamense* (gloeosporioides complex) was also common in Indonesia, Sri Lanka and Thailand. Pathogenicity tests on chili fruit showed that *C. javanense* and *C. scovillei* were highly aggressive, especially when inoculated on non-wounded fruit, compared to all other species. The existence of new, highly aggressive exotic species, such as *C. javanense*, poses a biosecurity risk to production in countries which do not have adequate quarantine regulations to restrict the entry of exotic pathogens.

## INTRODUCTION

*Colletotrichum* is one of the most important genera of plant pathogenic fungi with many of the 200 plus species known to cause disease in plant crops worldwide (Udayanga et al. 2013; Marin-Felix et al. 2017). *Colletotrichum* species causing anthracnose are particularly important as post-harvest pathogens of fruit and vegetable crops growing in tropical and subtropical climates (Alahakoon et al. 1994; Rojas et al. 2010; Cannon et al. 2012; Damm et al. 2012a, 2012b; Udayanga et al. 2013; Shivas et al. 2016; De Silva et al. 2017a).

Chili (*Capsicum* spp.) is an important vegetable crop in tropical and subtropical climates and the fresh or dried fruit is a major culinary ingredient in many cuisines. Anthracnose is a major disease of chili fruit causing significant yield loss as well as reducing the marketability of the fruit. Anthracnose of chili has been shown to be caused by 24 *Colletotrichum* species (Mongkolporn and Taylor 2018) reported from many countries including Australia (De Silva et al. 2017a), Brazil (de Oliveira et al. 2017), China (Diao et al. 2017), India (Sharma and Shenoy 2014), Indonesia (Voorrips et al. 2004), Korea (Kim et al. 1999), Malaysia (Noor and Zakaria 2018), Sri Lanka (Ranathunge et al. 2009), Thailand (Than et al. 2008) and the USA (Harp et al. 2008).

*Colletotrichum* species causing anthracnose of chili in Asia were previously identified as *C. acutatum* (straight conidia with acute ends), *C. gloeosporioides* (straight conidia with obtuse ends) and *C. truncatum* (falcate conidia) (Than et al. 2008, Mongkolporn et al. 2010,). However, with the implementation of multigene phylogenetic analyses, *C. acutatum* was demonstrated to be a species complex (acutatum complex) composed of 34 closely related species (Marin-Felix et al. 2017), with seven species identified as causing anthracnose in chili (Mongkolporn and Taylor 2018). Similarly, *C. gloeosporioides* was shown to be a species complex of 38 closely related species (Marin-Felix et al. 2017), with nine species identified to cause anthracnose in chili (Mongkolporn and Taylor 2018). Morphological characters cannot differentiate many of the species with straight conidia, especially those in the acutatum and gloeosporioides complexes that are pathogens of chili, and thus multigene phylogenetic analyses are required for proper identification of these species (De Silva et al. 2017a).

The distribution of the *Colletotrichum* species that cause anthracnose of chili is quite variable across countries that produce this crop. For example, in Australia only six out of the 24 *Colletotrichum* pathogens of chili have been identified (De Silva et al. 2017a), in Thailand only three have been reported (Mongkolporn and Taylor 2018), and five from Malaysia (Noor and Zakaria 2018). In most of the previous reports the identification of species was based only on morphological data. Therefore, the status of the taxonomy of *Colletotrichum* spp. causing anthracnose in chili producing countries in Asia remains uncertain. Proper identification of these pathogens is important for mitigating the risk of incursion of new pathogens which if happens, may have devastating consequences for the local industries. In addition, accurate identification of the species is important for resistance breeding programs and in identifying the host-range of species. Several *Colletotrichum* species such as *C. karsti, C. siamense* and *C. truncatum* have broad host ranges (Cannon et al. 2012; Damm et al. 2012b). The main *Colletotrichum* species causing anthracnose in chili are known to be in the acutatum and gloeosporioides complexes. However, recently further species from the boninense and orchidearum complexes were implicated (Diao et al. 2017; Damm et al. 2019). Therefore, it is important to understand the taxonomy, diversity and pathogenicity of *Colletotrichum* species that infect chili and their distribution across countries.

The aims of the study were to (1) identify the *Colletotrichum* species with straight conidia (cylindrical and fusiform) causing anthracnose of chili in selected regions of Indonesia, Malaysia, Taiwan, Thailand and Sri Lanka, and (2) determine the pathogenicity of those species on chili.

## MATERIALS AND METHODS

### Isolates

A total of 260 isolates associated with anthracnose disease symptoms on chili fruit and leaves were collected from chili producing countries in Asia: Indonesia, Malaysia, Taiwan, Thailand, and Sri Lanka (Table 1). Type specimens and ex-type cultures are deposited in the Westerdijk Fungal Biodiversity Institute, Utrecht, The Netherlands (CBS), and in the University of Melbourne culture collection (UOM), Victoria, Australia.

**Table 1:** Collection sites and numbers of *Colletotrichum* isolates

Country and region	Number of isolates
**Thailand**	**96**
Chiang Mai	20
Chiang Rai	44
Kanchana Buri	4
Nakhon Pathom	7
Suphan Buri	12
Ratchaburi	7
Bangkok	2
**Malaysia**	**12**
Pahang	3
Johor	4
Kelantan	5
**Sri Lanka**	**19**
Kandy	5
Matara	14
**Indonesia**	**113**
Gowa	31
Soppeng	6
Jeneponto	45
Makassar	7
Maros	4
West Java (East West Seed Co. Indonesia)	20
**Taiwan** (World Vegetable Center collection)	**20**
Tainan	14
Taichung	1
Nantou	2
Pingtung	1
Ilan	1
Hsinchu	1

Figures in bold represent the total number of isolates from each country

Fungal isolates were established from lesions on infected fruit and leaves using two methods. Surface sterilised (~ 1% ai sodium hypochlorite for 5 min) infected tissue (0.5 cm^2^) was cultured on water agar (WA; Crous et al. 2009) and then after 2 to 3 d fungal hyphae were subcultured onto potato dextrose agar (PDA, Difco) and synthetic nutrient-poor agar (SNA, Nirenberg 1976) as described by De Silva et al. (2017a). Freshly collected fruit from field grown chili plants with typical anthracnose lesions was incubated for 1 to 2 d in a moist chamber until conidiomata appeared and then single spore isolation was performed according to Choi et al. (1999). Selected isolates were also cultured on oatmeal agar (OA; Crous et al. 2009) and malt extract agar (MEA) at 20 °C under near UV light with a 12 h photoperiod for 10 d. Cultures were isolated and maintained either at the AQIS quarantine laboratory at the University of Melbourne or the Evolutionary Pathology Laboratory at the Westerdijk Fungal Biodiversity Institute, Netherlands (CBS).

### Morphology

Cultures grown on PDA at 27 °C were used for morphological analysis. Colony colour and texture were examined after 10 d, and colony growth rate calculated by measuring colony diameter 7 and 10 d after incubation.

Conidia from the conidiomata in culture were mounted in lactic acid and the length and width measured for 30 randomly selected conidia for each isolate, with the range and mean calculated. Size and shape of appressoria were determined on WA using a slide culture technique (Johnston and Jones 1997). Production of acervular conidiomata was observed on dried, sterilised chili peduncles inoculated with mycelia and incubated on WA and SNA media. Cultures were examined periodically for the development of perithecia. Ascospores were measured and described from perithecia squashed in lactic acid. Morphological characters were examined using a Leica DM6000 LED compound microscope with differential interference contrast (DIC) optics.

### DNA extraction, PCR amplification and sequencing

The 260 *Colletotrichum* isolates were initially identified on the basis of culture characteristics on PDA (based on distinct morphotype groups), morphology of the spores, and/or internal transcribed spacer and intervening 5.8S nrDNA gene (ITS) sequence. A total of 115 isolates were identified as *C. truncatum* and the remaining 145 isolates with straight conidia were subsequently selected for multigene phylogenetic analyses. Genomic DNA was extracted from fresh mycelia grown on PDA using the DNeasy Plant Mini kit (QIAGEN, Australia), following the manufacturer’s instructions. DNA quality was assessed on a 1.4% (w/v) agarose gel, quantified by comparing with a known amount of Lambda DNA/*Hind*III marker (Invitrogen, Australia), diluted to 2 ng/μL and then stored at ^_^20 °C until ready for PCR.

Isolates belonging to the acutatum complex were further analysed with partial gene sequences of five genomic loci: an intron sequence of the glyceraldehyde-3-phosphate dehydrogenase (*gapdh*), partial sequences of the chitin synthase 1 (*chs-1*), actin (*act*), beta-tubulin (*tub2*) and histone 3 (*his3*) genes. Isolates of the gloeosporioides complex were further analysed with *chs-1, act, gapdh, tub2*, Apn2–MAT1–2 intergenic spacer and partial mating type MAT1–2 gene (*ApMat*) and glutamine synthetase (*gs*) genes. Isolates belonging to the boninense and orchidearum complexes were further analysed with *gapdh, tub2* and *act* genes. The genes were amplified and sequenced using the respective primer pairs for each gene region: ACT-512F + ACT-783R (*act*; Carbone and Kohn 1999), AMF1 + AMR1 (*ApMat*; Silva et al. 2012b), CHS-79F + CHS-345R (*chs-1*; Carbone and Kohn 1999), GDF1 + GDR1 (*gapdh*; Guerber et al. 2003), GSF1 + GSR1 (*gs*; Stephenson et al. 1997), CYLH3F + CYLH3R (*his3*; Crous et al. 2004a), ITS1 + ITS4 (ITS; White et al. 1990), and Btub2Fd + Btub4Rd (*tub2*; Woudenberg et al. 2009).

The PCR for each reaction was performed in a 2720 Thermal Cycler (Applied Biosystems) in a total volume of 25 μL, comprised of 1× PCR buffer, 0.2 mM dNTP, 0.4 μM of each primer, 2 mM MgCl_2_, 1 U Taq DNA polymerase (MangoTaq DNA polymerase; Bioline) and 6 ng template DNA and components were adjusted as required. PCR amplification protocols were performed as described by Damm et al. (2012a, 2012b) and Silva et al. (2012), except for the annealing temperatures which were adjusted to 55 °C for ITS*, gapdh, tub2*, 58 °C for *act*, 60 °C for *gs* and 62 °C for *ApMat*.

All PCR products were purified with the QIAquick PCR Purification kit (QIAGEN, Australia), according to manufacturer’s instructions. DNA sequence analysis of the PCR products was carried out at either the Australian Genome Research Facility (AGRF, Melbourne) or at the Westerdijk Fungal Biodiversity Institute, Utrecht, the Netherlands. The purified PCR products were sequenced in both forward and reverse directions, and the consensus sequences were obtained by alignment using Geneious Pro v. 11.1.4 (Kearse et al. 2012). The consensus sequences were deposited in GenBank (Table 2) and taxonomic novelties in MycoBank (Crous et al. 2004b). Sequences of each locus were assembled with MEGA v. 6 (Tamura et al. 2013). GenBank accession numbers of all the isolates used in the phylogenetic analyses are listed in Table 2.

**Table 2:** Strains of *Colletotrichum* species used in the phylogenetic analyses with details of host and location, and GenBank accession numbers of the sequences

Species	Accession No.1	Host/Substrate	Country	GenBank accession number							
				ITS	*gapdh*	*chs-1*	*his3*	*act*	*tub2*	*ApMat*	*gs*
Acutatum complex											
*C.abscissum*	COAD 1877^a^	*Citrus sinensis*	Brazil	KP843126	KP843129	KP843132	KP843138	KP843141	KP843135		
*C. acutatum*	CBS 112996, ATCC 56816, STE-U 5292^a^	*Carica papaya*	Australia	JQ005776	JQ948677	JQ005797	JQ005818	JQ005839	JQ005860	**–**	**–**
	CBS 144.29	*Capsicum annuum*	Sri Lanka	JQ948401	JQ948732	JQ949062	JQ949392	JQ949722	JQ950052	**–**	**–**
*C. australisinense*	CGMCC 3.18886, GX1655^a^	*Hevea brasiliensis*	China	MG209623	MG241962	MG241981	–	MG241947	MG209645		
*C. bannaense*	CGMCC 3.18887, YNWD31^a^	*Hevea brasiliensis*	China	MG209638	MG242006	MG241996	–	MG242002	MG209660		
*C. brisbanense*	CBS 292.67, DPI 11711^a^	*Capsicum annuum*	Australia	JQ948291	JQ948621	JQ948952	JQ949282	JQ949612	JQ949942	**–**	**–**
*C. cairnsense*	BRIP 63642a, CBS 140847^a^	*Capsicum annuum*	Australia	KU923672	KU923704	KU923710	KU923722	KU923716	KU923688	**–**	**–**
*C. chrysanthemi*	CBS 126518, PD 84/520^a^	*Carthamus* sp., twisted stem	Netherlands	JQ948271	JQ948601	JQ948932	JQ949262	JQ94992	JQ949922	**–**	**–**
* C. cosmi*	CBS 853.73, PD 73/856^a^	*Cosmos* sp., seed	Netherlands	JQ948274	JQ948604	JQ948935	JQ949265	JQ949595	JQ949925	**–**	**–**
*C. costaricense*	CBS 330.75^a^	*Coffea arabica*, cv. *Typica*, berry	Costa Rica	JQ948180	JQ948510	JQ948841	JQ949171	JQ949501	JQ949831		
*C. citri*	CBS 134233^a^	*C. aurantifolia* shoot	China	KC293581	KC293741	**–**	**–**	KC293621	KC293661		
*C. cuscutae*	IMI 304802^a^	*Cuscuta* sp.	Dominica	JQ948195	JQ948525	JQ948856	JQ949186	JQ949516	JQ949846		
*C. fioriniae*	CBS 128517^a^	*Fiorinia* sp.	USA	JQ948292	JQ948622	JQ948953	JQ949283	JQ949613	JQ949943	**–**	**–**
*C. godetiae*	CBS 133.44^a^	*Clarkia hybrida*, cv. *Kelvon Glory*, seed	Denmark	JQ948402	JQ948733	JQ949063	JQ949393	JQ949723	JQ950053	**–**	**–**
*C. guajavae*	IMI 350839, CPC 18893^a^	*Psidium guajava*, fruit	India	JQ948270	JQ948600	JQ948931	JQ949261	JQ949591	JQ949921	**–**	**–**
*C. indonesiense*	CBS 127551, CPC 14986^a^	*Eucalyptus sp.*	Indonesia	JQ948288	JQ948618	JQ948949	JQ949279	JQ949609	JQ949939	**–**	**–**
*C. javanense*	**CBS 144963** ^**a**^ **, UOM 1115, EWINDO 3**	***Capsicum annuum***	**Indonesia**	**MH846576**	**MH846572**	**MH846573**	**MH846571**	**MH846575**	**MH846574**	**–**	**–**
*C. laticiphilum*	CBS 112989, IMI 383015^a^	*Hevea brasiliensis*	India	JQ948289	JQ948619	JQ948950	JQ949280	JQ949610	JQ949940	**–**	**–**
*C. limetticola*	CBS 114.14^a^	*Citrus aurantifolia*, young twig	USA, Florida	JQ948193	JQ948523	JQ948854	JQ949184	JQ949514	JQ949844		
*C. lupini*	CBS 109225, BBA 70884^a^	*Lupinus albus*	Ukraine	JQ948155	JQ948485	JQ948816	JQ949146	JQ949476	JQ949806	**–**	**–**
*C. melonis*	CBS 159.84^a^	*Cucumis melo*	Brazil	JQ948194	JQ948524	JQ948855	JQ949185	JQ949515	JQ949845		
*C. nymphaeae*	CBS 515.78^a^	*Nymphaea alba*, leaf spot	Netherlands	JQ948197	JQ948527	JQ948858	JQ949518	JQ949848	JQ949848	**–**	**–**
*C. paranaense*	CBS 134729^a^, CPC 20901	*Malus domestica*	Brazil	KC204992	KC205026	KC205043	KC205004	KC205077	KC205060		
*C. paxtonii*	IMI 165753^a^, CPC 18868	*Musa* sp.	Saint Lucia	JQ948285	JQ948615	JQ948946	JQ949276	JQ949606	JQ949936	**–**	**–**
*C. salicis*	CBS 607 94^a^	*Salix sp.*, leaf spot	Netherlands	JQ948460	JQ948791	JQ949121	JQ949451	JQ949781	JQ950111	**–**	**–**
*C. scovillei*	CBS 120708, HKUCC 10893, Mj6	*Capsicum annuum*	Thailand	JQ948269	JQ948599	JQ948930	JQ949260	JQ949590	JQ949920	**–**	**–**
	CBS 126529, PD 94/921–3, BBA 70349^a^	*Capsicum* sp.	Indonesia	JQ948267	JQ948597	JQ948928	JQ949258	JQ949588	JQ949918	**–**	**–**
	**CPC 28551**	***Capsicum annuum***	**Thailand**	**MH618287**	**MH618361**	**MH686337**	**MH707595**	**MH645871**	**–**	**–**	**–**
	**CPC 28552**	***Capsicum annuum***	**Thailand**	**MH618286**	**MH618362**	**MH686338**	**MH707594**	**MH645872**	**–**	**–**	**–**
	**CPC 28577**	***Capsicum annuum***	**Indonesia**	**MH618295**	**MH618363**	**MH686339**	**MH707593**	**MH645873**	**–**	**–**	**–**
	**CPC 28579**	***Capsicum annuum***	**Indonesia**	**MH618294**	**MH618364**	**MH686340**	**MH707592**	**MH645874**	**–**	**–**	**–**
	**CPC 28591**	***Capsicum annuum***	**Indonesia**	**MH618293**	**MH618365**	**MH686341**	**MH707591**	**MH645875**	**–**	**–**	**–**
	**CPC 28593**	***Capsicum annuum***	**Indonesia**	**MH618292**	**MH618366**	**MH686342**	**MH707590**	**MH645876**	**–**	**–**	**–**
	**CPC 28599**	***Capsicum annuum***	**Indonesia**	**MH618291**	**MH618367**	**MH686343**	**MH707589**	**MH645877**	**–**	**–**	**–**
	**CPC 28603**	***Capsicum annuum***	**Indonesia**	**MH618290**	**MH618368**	**MH686344**	**MH707588**	**MH645878**	**–**	**–**	**–**
	**CPC 28615**	***Capsicum annuum***	**Indonesia**	**MH618289**	**MH618369**	**MH686345**	**MH707587**	**MH645879**	**–**	**–**	**–**
	**CPC 28617**	***Capsicum annuum***	**Indonesia**	**MH618288**	**MH618370**	**MH686346**	**MH707586**	**MH645880**	**–**	**–**	**–**
	**CPC 30197, Coll 1**	***Capsicum annuum***	**Indonesia**	**MH618268**	**MH618334**	**MH686347**	**MH707585**	**MH645881**	**–**	**–**	**–**
	**CPC 30198, Coll 2**	***Capsicum annuum***	**Indonesia**	**MH618269**	**MH618335**	**MH686348**	**MH707584**	**MH645882**	**–**	**–**	**–**
	**CPC 30199, Coll 3**	***Capsicum annuum***	**Indonesia**	**MH618270**	**MH618336**	**MH686349**	**MH707583**	**MH645883**	**–**	**–**	**–**
	**CPC 30200, Coll 4**	***Capsicum annuum***	**Indonesia**	**MH618271**	**MH618337**	**MH686350**	**MH707582**	**MH645884**	**–**	**–**	**–**
	**CPC 30201, Coll 5**	***Capsicum annuum***	**Indonesia**	**MH618272**	**MH618338**	**MH686351**	**MH707581**	**MH645885**	**–**	**–**	**–**
	**CPC 30202, Coll 6**	***Capsicum annuum***	**Indonesia**	**MH618273**	**MH618339**	**MH686352**	**MH707580**	**MH645886**	**–**	**–**	**–**
	**CPC 30205, Coll 9**	***Capsicum annuum***	**Indonesia**	**MH618274**	**MH618340**	**MH686353**	**MH707579**	**MH645887**	**–**	**–**	**–**
	**CPC 30206, Coll 10**	***Capsicum annuum***	**Indonesia**	**MH618275**	**MH618341**	**MH686354**	**MH707578**	**MH645888**	**–**	**–**	**–**
	**CPC 30215, Coll 19**	***Capsicum annuum***	**Indonesia**	**MH618276**	**MH618342**	**MH686355**	**MH707577**	**MH645889**	**–**	**–**	**–**
	**CPC 30216, Coll 20**	***Capsicum annuum***	**Indonesia**	**MH618277**	**MH618343**	**MH686356**	**MH707576**	**MH645890**	**–**	**–**	**–**
	**CPC 30217, Coll 21**	***Capsicum annuum***	**Indonesia**	**MH618278**	**MH618344**	**MH686357**	**MH707575**	**MH645891**	**–**	**–**	**–**
	**CPC 30218, Coll 22**	***Capsicum annuum***	**Indonesia**	**MH618279**	**MH618345**	**MH686358**	**MH707574**	**MH645892**	**–**	**–**	**–**
	**CPC 30219, Coll 23**	***Capsicum annuum***	**Indonesia**	**MH618280**	**MH618346**	**MH686359**	**MH707573**	**MH645893**	**–**	**–**	**–**
	**CPC 30220, Coll 24**	***Capsicum annuum***	**Indonesia**	**MH618281**	**MH618347**	**MH686360**	**MH707572**	**MH645894**	**–**	**–**	**–**
	**CPC 30229, Coll 33**	***Capsicum annuum***	**Thailand**	**MH618282**	**MH618348**	**MH686361**	**MH707571**	**MH645895**	**–**	**–**	**–**
	**CPC 30230, Coll 34**	***Capsicum annuum***	**Thailand**	**MH618283**	**MH618349**	**MH686362**	**MH707570**	**MH645896**	**–**	**–**	**–**
	**CPC 30231, Coll 35**	***Capsicum annuum***	**Thailand**	**MH618284**	**MH618350**	**MH686363**	**MH707569**	**MH645897**	**–**	**–**	**–**
	**CPC 30232, Coll 36**	***Capsicum annuum***	**Thailand**	**MH618285**	**MH618351**	**MH686364**	**MH707568**	**MH645898**	**–**	**–**	**–**
	**CPC 30239, Coll 153**	***Capsicum annuum***	**Taiwan**	**MH618299**	**MH836634**	**MH707528**	**MH707611**	**MH645855**	**MH635064**	**–**	**–**
	**CPC 30240, Coll 329**	***Capsicum annuum***	**Taiwan**	**MH618300**	**MH836635**	**MH707529**	**MH707610**	**MH645856**	**MH635065**	**–**	**–**
	**CPC 30241, Coll 524**	***Capsicum annuum***	**Taiwan**	**MH618301**	**MH836637**	**MH707530**	**MH707609**	**MH645857**	**MH635067**	**–**	**–**
	**CPC 30242, Coll 683**	***Capsicum annuum***	**Taiwan**	**MH618302**	**MH836638**	**MH707531**	**MH707608**	**MH645858**	**MH635068**	**–**	**–**
	**CPC 30243, Coll 1296**	***Capsicum annuum***	**Taiwan**	**MH618303**	**MH836639**	**MH707532**	**MH707607**	**MH645859**	**MH635069**	**–**	**–**
	**CPC 30244, Coll 1297**	***Capsicum annuum***	**Taiwan**	**MH618304**	**MH836640**	**MH707533**	**MH707606**	**MH645860**	**MH635070**	**–**	**–**
	**CPC 30246, Coll 1300**	***Capsicum annuum***	**Taiwan**	**MH618305**	**MH836641**	**MH707534**	**MH707605**	**MH645861**	**MH635071**	**–**	**–**
	**CPC 30247, Coll 1301**	***Capsicum annuum***	**Taiwan**	**MH618306**	**MH836642**	**MH707535**	**MH707604**	**MH645862**	**MH635072**	**–**	**–**
	**CPC 30248, Coll 1302**	***Capsicum annuum***	**Taiwan**	**MH618308**	**MH836643**	**MH707536**	**MH707603**	**MH645863**	**MH635073**	**–**	**–**
	**CPC 30249, Coll 1303**	***Capsicum annuum***	**Taiwan**	**MH618307**	**MH836644**	**MH707537**	**MH707602**	**MH645864**	**MH635074**	**–**	**–**
	**CPC 30250, Coll 1304**	***Capsicum annuum***	**Taiwan**	**MH618309**	**MH836645**	**MH707538**	**MH707601**	**MH645865**	**MH635075**	**–**	**–**
	**CPC 30251, Coll 1306**	***Capsicum annuum***	**Taiwan**	**MH618310**	**MH836646**	**MH707539**	**MH707600**	**MH645866**	**MH635076**	**–**	**–**
	**CPC 30252, Coll 141**	***Capsicum annuum***	**Taiwan**	**MH618311**	**MH836633**	**MH707540**	**MH707599**	**MH645867**	**MH635063**	**–**	**–**
	**UOM 1101, 313**	***Capsicum annuum***	**Thailand**	**MH618256**	**MH618324**	**MH686324**	**MH707557**	**MH635089**	**MH635049**	**–**	**–**
	**UOM 1102, 322**	***Capsicum annuum***	**Thailand**	**MH618259**	**MH618325**	**MH686325**	**MH707556**	**MH635090**	**MH635050**	**–**	**–**
	**UOM 1103, 311**	***Capsicum annuum***	**Thailand**	**MH618255**	**MH618326**	**MH686326**	**MH707555**	**MH635091**	**MH635051**	**–**	**–**
	**UOM 1104, 314**	***Capsicum annuum***	**Thailand**	**MH618257**	**MH618327**	**MH686327**	**MH707554**	**MH635092**	**MH635052**	**–**	**–**
	**UOM 1105, MJ3**	***Capsicum annuum***	**Thailand**	**MH618264**	**MH618328**	**MH686328**	**MH707553**	**MH635093**	**MH635053**	**–**	**–**
	**UOM 1106, MJ5**	***Capsicum annuum***	**Thailand**	**MH618265**	**MH618329**	**MH686329**	**MH707552**	**MH635094**	**MH635054**	**–**	**–**
	**UOM 1107, MJ7**	***Capsicum annuum***	**Thailand**	**MH618266**	**MH618330**	**MH686330**	**MH707551**	**MH635095**	**MH635055**	**–**	**–**
	**UOM 1108, MJ8**	***Capsicum annuum***	**Thailand**	**MH618267**	**MH618331**	**MH686331**	**MH707550**	**MH635096**	**MH635056**	**–**	**–**
	**UOM 1109, 211**	***Capsicum annuum***	**Thailand**	**MH618254**	**MH618332**	**MH686332**	**MH707549**	**MH635097**	**MH635057**	**–**	**–**
	**UOM 1110, 316**	***Capsicum annuum***	**Thailand**	**MH618258**	**MH618333**	**MH686333**	**MH707548**	**MH635098**	**MH635058**	**–**	**–**
	**UOM 1111, GA1**	***Capsicum annuum***	**Thailand**	**MH618260**	**MH618357**	**MH686334**	**MH707547**	**MH635099**	**MH635059**	**–**	**–**
	**UOM 1112, GA2**	***Capsicum annuum***	**Thailand**	**MH618261**	**MH618358**	**MH686335**	**MH707546**	**MH635100**	**MH635060**	**–**	**–**
	**UOM 1113, GA3**	***Capsicum annuum***	**Thailand**	**MH618262**	**MH618359**	**–**	**MH707545**	**MH635101**	**MH635061**	**–**	**–**
	**UOM 1114, GA5**	***Capsicum annuum***	**Thailand**	**MH618263**	**MH618360**	**MH686336**	**MH707544**	**MH635102**	**MH635062**	**–**	**–**
	**UOM 1140, F59**	***Capsicum annuum***	**Malaysia**	**MH618316**	**MH618355**	**MH686322**	**MH707559**	**MH635087**	**MH635047**	**–**	**–**
	**UOM 1141, A15**	***Capsicum annuum***	**Malaysia**	**MH618313**	**MH618356**	**MH686323**	**MH707558**	**MH635088**	**MH635048**	**–**	**–**
	**UOM 1142, Coll 1307**	***Capsicum annuum***	**Taiwan**	**MH618298**	**MH836647**	**MH707542**	**MH707597**	**MH645869**	**MH635077**	**–**	**–**
	**UOM 1143, Coll 1311**	***Capsicum annuum***	**Taiwan**	**MH618296**	**MH836648**	**MH707543**	**MH707596**	**MH645870**	**MH635078**	**–**	**–**
	**UOM 1144, EWINDO 2**	***Capsicum annuum***	**Indonesia**	**MH587232**	**MH618317**	**MH686314**	**MH707567**	**MH635079**	**MH836628**	**–**	**–**
	**UOM 1145, EWINDO 8**	***Capsicum annuum***	**Indonesia**	**MH587231**	**MH618318**	**MH686315**	**MH707566**	**MH635080**	**MH836629**	**–**	**–**
	**UOM 1146, EWINDO 10**	***Capsicum annuum***	**Indonesia**	**MH587233**	**MH618319**	**MH686316**	**MH707565**	**MH635081**	**MH836630**	**–**	**–**
	**UOM 1147, EWINDO 14**	***Capsicum annuum***	**Indonesia**	**MH587234**	**MH618320**	**MH686317**	**MH707564**	**MH635082**	**MH836631**	**–**	**–**
	**UOM 1148, EWINDO 15**	***Capsicum annuum***	**Indonesia**	**MH587235**	**MH618321**	**MH686318**	**MH707563**	**MH635083**	**MH836632**	**–**	**–**
	**UOM 1149, Coll 365**	***Capsicum annuum***	**Taiwan**	**MH618297**	**MH836636**	**MH707541**	**MH707598**	**MH645868**	**MH635066**	**–**	**–**
	**UOM 1150, 4–46-3D**	***Capsicum annuum***	**Malaysia**	**MH618312**	**MH618352**	**MH686319**	**MH707562**	**MH635084**	**MH635044**	**–**	**–**
	**UOM 1151, E15**	***Capsicum annuum***	**Malaysia**	**MH618314**	**MH618353**	**MH686320**	**MH707561**	**MH635085**	**MH635045**	**–**	**–**
	**UOM 1152, E16**	***Capsicum annuum***	**Malaysia**	**MH618315**	**MH618354**	**MH686321**	**MH707560**	**MH635086**	**MH635046**	**–**	**–**
*C. simmondsii*	CBS 122122^a^	*Carica papaya,* fruit	Australia	JQ948276	JQ948606	JQ948937	JQ949267	JQ949597	JQ949927	**–**	**–**
*C. sloanei*	IMI 364297, CPC 18929^a^	*Theobroma cacao,* leaf	Malaysia	JQ948287	JQ948617	JQ948948	JQ949278	JQ949608	JQ949938	**–**	**–**
*C. tamarilloi*	CBS 129814, T.A.6^a^	*Solanum betaceum, *fruit	Colombia	JQ948184	JQ948514	JQ948845	JQ949175	JQ949505	JQ949835	**–**	**–**
*C. walleri*	CBS 125472, BMT(HL)19^a^	*Coffea *sp., leaf tissue	Vietnam	JQ948275	JQ948605	JQ948936	JQ949266	JQ949596	JQ949926	**–**	**–**
Boninense complex											
*C. annellatum*	CBS 129826^a^	*Hevea brasiliensis,* leaf	Colombia	JQ005222	JQ005309	JQ005396	–	JQ005570	JQ005656	**–**	**–**
*C. beeveri*	CBS 128527, ICMP 18594^a^	*Brachyglottis repanda*	New Zealand	JQ005171	JQ005258	JQ005345	–	JQ005519	JQ005605	**–**	**–**
* C. boninense*	CBS 123755^a^, MAFF 305972	*Crinum asiaticum *var. *sinicum*	Japan	JQ005153	JQ005240	JQ005327	–	JQ005501	JQ005588	**–**	**–**
*C. brasiliense*	CBS 128501^a^, ICMP 18607, PAS12	*Passiflora edulis*, fruit anthracnose	Brazil	JQ005235	JQ005322	JQ005409	–	JQ005583	JQ005669	**–**	**–**
*C. brassicicola*	CBS 101059^a^, LYN 16331	*Brassica oleracea *var. *gemmifera*, leaf spot	New Zealand	JQ005172	JQ005259	JQ005346	–	JQ005520	JQ005606	**–**	**–**
*C. constrictum*	CBS 128504^a^, ICMP 12941	*Citrus limon*, fruit rot	New Zealand	JQ005238	JQ005325	JQ005412	–	JQ005586	JQ005672	**–**	**–**
*C. karsti*	CAUOS1	*Capsicum *sp.	China	KP890103	KP890134	KP890118	–	KP890126	KP890110	**–**	**–**
	CAUOS7	*Capsicum* sp.	China	KP890108	KP890139	KP890124	–	KP890132	KP890116	**–**	**–**
	CBS 125468	*Coffea *sp., berry tissue	Vietman	JQ005197	JQ005284	JQ005371	–	JQ005545	JQ005631	**–**	**–**
	CBS 127595	*Musa banksii*	Australia	JQ005178	JQ005265	JQ005352	–	JQ005526	JQ005612	**–**	**–**
	CBS 129815, T.A.7	*Solanum betaceum*, fruit	Colombia	JQ005187	JQ005274	JQ005361	–	JQ005535	JQ005621	**–**	**–**
	CBS 129834	*Musa *sp.	Mexico	JQ005176	JQ005263	JQ005350	–	JQ005524	JQ005610	**–**	**–**
	CBS 129927	*Anthurium *sp.	Thailand	JQ005206	JQ005293	JQ005380	–	JQ005554	JQ005640	**–**	**–**
	CBS 128545, ICMP 18587	*Capsicum annuum*	New Zealand	JQ005207	JQ005294	JQ005381	–	JQ005555	JQ005641	**–**	**–**
	CBS 128548, ICMP 18589	*Solanum lycopersicum*	New Zealand	JQ005205	JQ005292	JQ005379	–	JQ005553	JQ005639	**–**	**–**
	CBS 132134, CGMCC 3.14194^a^	*Vanda* sp.	China	HM585409	HM585391	–	–	HM581995	HM585428	**–**	**–**
	**CPC 28553**	***Capsicum annuum***	**Indonesia**	**MH844440**	**MH844444**	**MH844456**	–	**MH844449**	**MH844448**	**–**	**–**
	**CPC 28554**	***Capsicum annuum***	**Indonesia**	**MH844439**	**MH844443**	**MH844455**	–	**MH844450**	**MH844447**	**–**	**–**
	**CPC 28601**	***Capsicum annuum***	**Indonesia**	**MH844438**	**MH844442**	**MH844454**	–	**MH844451**	**MH844446**	**–**	**–**
	**CPC 28602**	***Capsicum annuum***	**Indonesia**	**MH844437**	**MH844441**	**MH844453**	–	**MH844452**	**MH844445**	**–**	**–**
	GM44 L01^a^	*Annona muricata*	Colombia	KC512141	KC506413	KC512162	–	KC512183	KC512204	**–**	**–**
*C. petchii*	CBS 378.94^a^	*Dracaena marginata*	Italy	JQ005223	JQ005310	JQ005397	–	JQ005571	JQ005657	**–**	**–**
*C. phyllanthi*	CBS 175.67^a^, MACS 271	*Phyllanthus acidus*	India	JQ005221	JQ005308	JQ005395	–	JQ005569	JQ005655	**–**	**–**
Truncatum complex											
*C. truncatum*	CBS 151.35	*Phaseolus lunatus*	USA	GU227862	GU228254	GU228352	–	GU227960	GU228156	**–**	**–**
Gloeosporioides complex											
*C. aenigma*	ICMP 18608^a^	*Persea americana*	Israel	JX010244	JX010044	JX009774	–	JX009443	JX010389	KM360143	JX010078
	ICMP 18686	*Pyrus pyrifolia*	Japan	JX010243	JX009913	JX009789	–	JX009519	JX010390	–	JX010079
*C. aeschynomenes*	ICMP 17673^a^, ATCC 201874	*Aeschynomene virginica*	USA	JX010176	JX009930	JX009799	–	JX009483	JX010392	KM360145	JX010081
*C. alatae*	CBS 304.67^a^, ICMP 17919	*Dioscorea alata*	India	JX010190	JX009990	JX009837	–	JX009471	JX010383	KC888932	JX010065
*C. alienum*	ICMP 12071^a^	*Malus domestica*	New Zealand	JX010251	JX010028	JX009882	–	JX009572	JX010411	KM360144	JX010101
	ICMP 18621	*Persea americana*	New Zealand	JX010246	JX009959	JX009755	–	JX009552	JX010386	–	JX010075
*C. aotearoa*	ICMP 18537^a^	*Coprosma *sp.	New Zealand	JX010205	JX010005	JX009853	–	JX009564	JX010420	KC888930	JX010113
*C. asianum*	ICMP 18580^a^, CBS 130418	*Coffea arabica*	Thailand	FJ972612	JX010053	JX009867	–	JX009584	JX010406	FR718814	JX010096
	IMI 313839, ICMP 18696	*Mangifera indica*	Australia	JX010192	JX009915	JX009753	–	JX009576	JX010384	–	JX010073
*C. camelliae*	CGMCC 3.14925, LC1364^a^	*Camellia sinensis*	China	KJ955081	KJ954782	–	–	KJ954363	KJ955230	KJ954497	KJ954932
*C. changpingense*	MFLUCC 150022^a^	*Fragaria ananassa*	China	KP683152	KP852469	KP852449	–	KP683093	KP852490	–	–
*C. chrysophilum*	CMM 4268^a^, URM 7362	*Musa *sp.	Brazil	KX094252	KX094183	KX094083	–	KX093982	KX094285	–	–
*C. clidemiae*	ICMP 18706^a^	*Clidemia hirta*	USA, Hawaii	JX010265	JX009989	JX009877	–	JX009537	JX010438	KC888929	JX010129
*C. conoides*	CAUG17^a^	*Capsicum annuum*	China	KP890168	KP890162	KP890156	–	KP890144	KP890174	–	–
*C. cordylinicola*	MFLUCC 090551^a^, ICMP 18579	*Cordyline fruticosa*	Thailand	JX010226	JX009975	JX009864	–	HM470235	JX010440	JQ899274	JX010122
*C. endophyticum*	CAUG28	*Capsicum annuum*	China	KP145441	KP145413	KP145385	–	KP145329	KP145469	–	–
	DNCL075	Unknown wild fruit	Thailand	KF242123	KF242181	–	–	KF157827	KF254857	–	KF242154
	LC0324^a^	*Pennisetum purpureum*	Thailand	KC633854	KC832854	–	–	KF306258	–	–	–
	**UOM 1137, F5-2D**	***Capsicum annuum***	**Thailand**	**MH728809**	**MH707467**	**MH805853**	–	**MH781483**	**MH846566**	**MH728828**	**MH748267**
*C. fructicola*	I TCC 6270	*Mangifera indica*	India	–	KC790774	KC888935	–	KC790663	KC790909	KC790713	–
	ICMP 18581^a^, CBS 130416	*Coffea arabica*	Thailand	JX010165	JX010033	JX009866	–	FJ907426	JX010405	JQ807838	JX010095
	LC2923, LF130	*Camellia sinensis*	China	KJ955083	KJ954784	–	–	KJ954365	KJ955232	KJ954499	KJ954934
	**CPC 28644**	***Capsicum annuum***	**Thailand**	**MH728811**	**MH707465**	**MH805851**	–	**MH781481**	**MH846564**	**MH728830**	**MH748265**
	**CPC 28645**	***Capsicum annuum***	**Thailand**	**MH728810**	**MH707466**	**MH805852**	–	**MH781482**	**MH846565**	**MH728829**	**MH748266**
	**UOM 1138, CPC 30253, Coll 853**	***Capsicum annuum***	**Taiwan**	**MH728817**	**MH707463**	**MH805846**	–	**MH781476**	**MH846559**	**MH728835**	**MH748260**
	**UOM 1139, Coll 1318**	***Capsicum annuum***	**Taiwan**	**MH728808**	**MH707468**	**MH805854**	–	**MH781484**	**MH846567**	**MH728827**	**MH748268**
*C. gloeosporioides*	IMI 356878, ICMP 17821, CBS 112999^a^	*Citrus sinensis*	Italy	JX010152	JX010056	JX009818	–	JX009531	JX010445	JQ807843	JX010085
*C. grevilleae*	CBS 132879, CPC 15481^a^	*Grevillea *sp.	Italy	KC297078	KC297010	KC296987	–	KC296941	KC297102	–	KC297033
*C. grossum*	CGMCC3.17614, CAUG7^a^	*Capsicum *sp.	China	KP890165	KP890159	KP890153	–	KP890141	KP890171	–	MG826120
*C. hebeiense*	MFLUCC13 0726^a^, JZB330028	*Vitis vinifera*	China	KF156863	KF377495	KF289008	–	KF377532	KF288975	–	–
*C. helleniense*	CBS 142418^a^, CPC 26844	*Poncirus trifoliata*	Greece	KY856446	KY856270	KY856186	–	KY856019	KY856528	–	–
*C. henanense*	LC3030, CGMCC 3.17354^a^	*Camellia sinensis*	China	KJ955109	KJ954810	–	–	KM023257	KJ955257	KJ954524	KJ954960
*C. horii*	ICMP 10492, MTCC 10841^a^	*Diospyros kaki*	Japan	GQ329690	GQ329681	JX009752	–	JX009438	JX010450	JQ807840	JX010137
*C. hystricis*	CBS 142411^a^, CPC 28153	*Citrus hystrix*	Italy	KY856450	KY856274	KY856190	–	KY856023	KY856532	–	–
*C. jiangxiense*	CGMCC 3.17363^a^	*Camellia sinensis*	China	KJ955201	KJ954902		–	KJ954471	KJ955348	KJ954607	KJ955051
*C. kahawae *subsp. *kahawae*	IMI 319418, ICMP 17816^a^	*Coffea arabica*	Kenya	JX010231	JX010012	JX009813	–	JX009452	JX010444	JQ894579	JX010130
***C. makassarense***	**CPC 28555**	***Capsicum annuum***	**Indonesia**	**MH728816**	**MH728822**	**MH805847**	–	**MH781477**	**MH846560**	**MH728834**	**MH748261**
	**CPC 28556**	***Capsicum annuum***	**Indonesia**	**MH728815**	**MH728821**	**MH805848**	–	**MH781478**	**MH846561**	**MH728833**	**MH748262**
	**CBS 143664** ^**a**^ **, CPC 28612**	***Capsicum annuum***	**Indonesia**	**MH728812**	**MH728820**	**MH805850**	–	**MH781480**	**MH846563**	**MH728831**	**MH748264**
*C. musae*	CBS 116870, ICMP 19119, MTCC 11349^a^	*Musa *sp.	USA	JX010146	JX010050	JX009896	–	JX009433	HQ596280	KC888926	JX010103
	CMM 4458	*Musa *sp.	Brazil	KX094249	KX094191	KX094080		KX093967	KX094292	–	–
*C. nupharicola*	CBS 469.96, ICMP 17938	*Nuphar lutea *subsp. *polysepala*	USA	JX010189	JX009936	JX009834	–	JX009486	JX010397	–	JX010087
	CBS 470.96, ICMP 18187^a^	*Nuphar lutea *subsp. *polysepala*	USA	JX010187	JX009972	JX009835	–	JX009437	JX010398	JX145319	JX010088
*C. perseae*	GA100^a^	*Persea americana*	Israel	KX620308	KX620242	–		KX620145	KX620341	KX620177	KX620275
*C. proteae*	CBS 132882^a^	*Proteaceae*	South Africa	KC297079	KC297009	KC296986		KC296940	KC297101		
*C. psidii*	CBS 145.29, ICMP 19120^a^	*Psidium* sp.	Italy	JX010219	JX009967	JX009901	–	JX009515	JX010443	KC888931	JX010133
*C. queenslandicum*	ICMP 1778^a^	*Carica papaya*	Australia	JX010276	JX009934	JX009899	–	JX009447	JX010414	KC888928	JX010104
	ICMP 18705	*Coffea* sp.	Fiji	JX010185	JX010036	JX009890	–	JX009490	JX010412		JX010102
	BRIP 63695	*Capsicum annuum*	Australia							KU923727	KU923737
*C. salsolae*	CBS 119296, ICMP 18693	*Glycine max* (inoculated)	Hungary	JX010241	JX009917	JX009791	–	JX009559	–	–	–
	ICMP 19051^a^	*Salsola tragus*	Hungary	JX010242	JX009916	JX009863	–	JX009562	JX010403	KC888925	JX010093
*C. siamense*	**CPC 28609**	***Capsicum annuum***	**Indonesia**	**MH728813**	**–**	**–**	–	**–**	**–**	**MH713886**	**MH748242**
	**CPC 30209, UOM 13**	***Capsicum annuum***	**Indonesia**	**MH707471**	**MH707452**	**MH805834**	–	**MH781464**	**MH846547**	**MH713897**	**MH748231**
	**CPC 30210, UOM 14**	***Capsicum annuum***	**Indonesia**	**MH707472**	**MH707453**	**MH805835**	–	**MH781465**	**MH846548**	**MH713896**	**MH748232**
	**CPC 30211, UOM15**	***Capsicum annuum***	**Indonesia**	**MH707473**	**MH707454**	**MH805836**	–	**MH781466**	**MH846549**	**MH713895**	**MH748233**
	**CPC 30212, UOM 16**	***Capsicum annuum***	**Indonesia**	**MH707474**	**MH707455**	**MH805837**	–	**MH781467**	**MH846550**	**MH713894**	**MH748234**
	**CPC 30221, UOM 25**	***Capsicum annuum***	**Thailand**	**MH707475**	**MH707456**	**MH805838**	–	**MH781468**	**MH846551**	**MH713893**	**MH748235**
	**CPC 30222, UOM26**	***Capsicum annuum***	**Thailand**	**MH707476**	**MH707457**	**MH805839**	–	**MH781469**	**MH846552**	**MH713892**	**MH748236**
	**CPC 30223, UOM27**	***Capsicum annuum***	**Thailand**	**MH707477**	**MH707458**	**MH805840**	–	**MH781470**	**MH846553**	**MH713891**	**MH748237**
	**CPC 30233, UOM37**	***Capsicum annuum***	**Indonesia**	**MH707478**	**MH707459**	**MH805841**	–	**MH781471**	**MH846554**	**MH713890**	**MH748238**
	**CPC 30234, UOM38**	***Capsicum annuum***	**Indonesia**	**MH707479**	**MH707460**	**MH805842**	–	**MH781472**	**MH846555**	**MH713889**	**MH748239**
	**CPC 30235, UOM39**	***Capsicum annuum***	**Indonesia**	**MH707480**	**MH707461**	**MH805843**	–	**MH781473**	**MH846556**	**MH713888**	**MH748240**
	**CPC 30236, UOM40**	***Capsicum annuum***	**Indonesia**	**MH707481**	**MH707462**	**MH805844**	–	**MH781474**	**MH846557**	**MH713887**	**MH748241**
	**UOM 1116**	***Capsicum annuum***	**Sri Lanka**	**MH707495**	**–**	**–**	–	**–**	**–**	**MH713872**	**MH748256**
	**UOM 1117**	***Capsicum annuum***	**Sri Lanka**	**MH707496**	**–**	**–**	–	**–**	**–**	**MH713871**	**MH748257**
	**UOM 1118**	***Capsicum annuum***	**Sri Lanka**	**MH707497**	**–**	**–**	–	**–**	**–**	**MH713870**	**MH748258**
	**UOM 1124, F1-3A**	***Capsicum annuum***	**Thailand**	**MH707482**	**–**	**–**	–	**–**	**–**	**MH713885**	**MH748243**
	**UOM 1125, F7-3B**	***Capsicum annuum***	**Thailand**	**MH707488**	**–**	**–**	–	**–**	**–**	**MH713879**	**MH748249**
	**UOM 1126, F4-1C**	***Capsicum annuum***	**Thailand**	**MH707484**	**–**	**–**	–	**–**	**–**	**MH713883**	**MH748245**
	**UOM 1127, F5-1A**	***Capsicum annuum***	**Thailand**	**MH707485**	**–**	**–**	–	**–**	**–**	**MH713882**	**MH748246**
	**UOM 1128, F7-1B**	***Capsicum annuum***	**Thailand**	**MH707487**	**–**	**–**	–	**–**	**–**	**MH713880**	**MH748248**
	**UOM 1129, F5-4A**	***Capsicum annuum***	**Thailand**	**MH707486**	**–**	**–**	–	**–**	**–**	**MH713881**	**MH748247**
	**UOM 1130, F1-3C**	***Capsicum annuum***	**Thailand**	**MH707483**	**–**	**–**	–	**–**	**–**	**MH713884**	**MH748244**
	**UOM 1131, F7-4A**	***Capsicum annuum***	**Thailand**	**MH707489**	**–**	**–**	–	**–**	**–**	**MH713878**	**MH748250**
	**UOM 1132, RC1**	***Capsicum annuum***	**Thailand**	**MH707490**	**–**	**–**	–	**–**	**–**	**MH713877**	**MH748251**
	**UOM 1133, RC2**	***Capsicum annuum***	**Thailand**	**MH707491**	**–**	**–**	–	**–**	**–**	**MH713876**	**MH748252**
	**UOM 1134, RC3**	***Capsicum annuum***	**Thailand**	**MH707492**	**–**	**–**	–	**–**	**–**	**MH713875**	**MH748253**
	**UOM 1135, RC4**	***Capsicum annuum***	**Thailand**	**MH707493**	**–**	**–**	–	**–**	**–**	**MH713874**	**MH748254**
	**UOM 1136, RC5**	***Capsicum annuum***	**Thailand**	**MH707494**	**–**	**–**	–	**–**	**–**	**MH713873**	**MH748255**
	IMI 82267, CPC 16808	*Vitis* sp.	Brazil							KP703783	KP703698
	ICMP 18575,HKUCC 10884	*Capsicum annuum*	Thailand	JX010256	JX010059	JX009785	–	JX009455	JX010404	KP703769	JX010094
	ICMP 18578^a^, CBS 130417	*Coffea arabica*	Thailand	JX010171	JX009924	JX009865		FJ907423	JX010404		JX010094
	LC0144, PE004–1	*Coffea* sp.	China, Yunnan							KP703785	KP703700
	LC0148, PE007–1	*Camellia* sp.	China, Yunnan							KJ954494	KJ954929
*C. siamense (*syn.* C. communis)*	NK24, MTCC 11599	*Mangifera indica*	India							JQ894582	
*C. siamense (*syn. *C. endomangiferae*)	CMM 3814^a^	*Mangifera indica*	Brazil	KC702994	KC702955	KC598113		KC702922	KM404170	KJ155453	
*C. siamense (*syn.* C. dianesei)*	CMM 4083	*Mangifera indica*	Brazil							KX094304	KX094216
	CMM 4085^a^	*Mangifera indica*	Brazil							KX094306	KX094218
*C. siamense (*syn. *C. hymenocallidis)*	CBS 125378, ICMP 18642, LC0043^a^	*Hymenocallis americana*	China	JX010278	JX010019	GQ856730	–	JX009441	JX010410	JQ899283	JX010100
	CBS 112983, CPC 2291	*Protea cynaroides*	Zimbabwe	KC297065	KC297007	KC296984	–	KC296929	KC297100	KP703761	KC297030
	CBS 113199. CPC 2290	*Protea cynaroides*	Zimbabwe	KC297066	KC297008	KC296985	–	KC296930	KC297090	KP703763	KC297031
	CBS 116868	*Musa *sp.	India; Southern India	KC566815	KC566669	KC566382	–	KC566961	KP703429	KP703764	KP703679
*C. siamense (*syn. *C. jasmini-sambac)*	CBS 130420^a^, ICMP 19118	*Jasminum sambac*	Vietnam	HM131511	HM131497	JX009895	–	HM131507	JX010415	JQ807841	JX010105
	CPC 16135, WTS9	*Persea americana*	South Africa	KP703760	KP703678	KC566375	–	KC566954	KP703597	KP703845	KP703760
*C. siamense (*syn. *C. melanocaulon)*	CBS 133251, coll131, BPI 884113^a^	*Vaccinium macrocarpon*	USA	**–**	**–**	–	–	–	–	JX145313	KP703685
*C. siamense (*syn.* C. murrayae)*	CBS 133239, GZAAS5.09506^a^	*Murraya *sp.	China	**–**	**–**	–	–	–	–	KP703770	JQ247621
*C. syzygicola*	DNCL021 MFLUCC 100624	*Syzygium samarangense*	Thailand	KF242094	KF242156	–	–	KF157801	KF254880	**–**	**–**
***C. tainanense***	**CBS 143666** ^a^ **, CPC 30245, UOM 1120, Coll 1298**	***Capsicum annuum***	**Taiwan**	**MH728818**	**MH728823**	**MH805845**	–	**MH781475**	**MH846558**	**MH728836**	**MH748259**
	**UOM 1119, Coll 1290**	***Capsicum annuum***	**Taiwan**	**MH728805**	**MH728819**	**MH805857**	–	**MH781487**	**MH846570**	**MH728824**	**MH748271**
*C. theobromicola*	MTCC 11350, CBS 124945, ICMP 18649^a^	*Theobroma cacao*	Panama	JX010294	JX010006	JX009869	–	JX009444	JX010447	KC790726	JX010139
*C. ti*	ICMP 4832^a^	*Cordyline* sp.	New Zealand	JX010269	JX009952	–	–	JX009520	JX010442	KM360146	JX010123
*C. tropicale*	CBS 124943, ICMP 18651	*Annona muricata*	Panama	JX010277	JX010014	JX009868	–	JX009570	–	KC790728	–
	CBS 124946	Unknown	Brazil	KC566806	KC566660	KC566373	–	KC566952	KC566228	**–**	**–**
	CBS 124949, ICMP 18653, MTCC 11371^a^	*Theobroma cacao*	Panama	JX010264	JX010007	JX009870	–	JX009489	JX010407	KC790728	JX010097
	CMM 4071	*Mangifera indica*	Brazil	KC329785	KC517181	–	–	KC533726	KC517258	**–**	**–**
	CMM 4243	*Musa *sp.	Brazil	KU213603	KU213601	KU213600	–	KU213596	KU213604	**–**	**–**
	CPC 16260	Unknown	Brazil	KC566807	KC566661	KC566374	–	KC566953	KC566229	**–**	**–**
	GM04-L01	*Annona muricata*	Colombia	KC512125	KC506397	KC512146	–	KC512167	KC512188	**–**	**–**
	GM33-L01	*Annona muricata*	Colombia	KC512128	KC506400	KC512149	–	KC512170	KC512191	**–**	**–**
	**CPC 28607**	***Capsicum annuum***	**Indonesia**	**MH728814**	**MH707464**	**MH805849**	–	**MH781479**	**MH846562**	**MH728832**	**MH748263**
	**UOM 1002**	***Capsicum annuum***	**Indonesia**	**MH728807**	**MH707469**	**MH805855**	–	**MH781485**	**MH846568**	**MH728826**	**MH748269**
	**UOM 1003**	***Capsicum annuum***	**Indonesia**	**MH728806**	**MH707470**	**MH805856**	–	**MH781486**	**MH846569**	**MH728825**	**MH748270**
*C. viniferum*	GZAAS 5.08601^a^	*Vitis vinifera*	China	JN412804	JN412798	–	–	JN412795	JN412813	**–**	JN412787
	CAUG27	*Capsicum* sp.	China	KP145440	KP145412	KP145356	–	KP145384	KP145468	**–**	**–**
*C. wuxiense*	CGMCC 3.17894^a^	*Camellia sinensis*	China	KU251591	KU252045	KU251939	–	KU251672	KU252200	KU251722	KU252101
*C. xanthorrhoeae*	BRIP 45094, ICMP 17903, CBS 127831^a^	*Xanthorrhoea preissii*	Australia	JX010261	JX009927	JX009823	–	JX009478	JX010448	KC790689	JX010138
Orchidearum complex											
*C. cattleyicola*	CBS 170.49^a^	*Cattleya* sp.	Belgium	MG600758	MG600819	–	–	MG600963	MG601025		
*C. cliviicola*	CBS 125375^a^	*Clivia miniata*	China	MG600733	MG600795	–	–	MG600939	MG601000	**–**	**–**
	CSSK4	*Clivia miniata*	China	GQ485607	GQ856756	–	–	GQ856777	GQ849440	**–**	**–**
	CSSS1	*Clivia miniata*	China	GU109479	GU085868	–	–	GU085861	GU085869	**–**	**–**
	CSSS2	*Clivia miniata*	China	GU109480	GU085868	–	–	GU085862	GU085870	**–**	**–**
*C. dracaenophilum*	CBS 118199^a^	*Dracaena sanderana*	China	JX519222	JX546707	–	–	JX519238	JX519247	**–**	**–**
*C. musicola*	CBS 132885^a^	*Musa* sp.	Mexico	MG600736	MG600798	–	–	MG600942	MG601003	**–**	**–**
*C. orchidearum*	CBS 135131^a^	*Dendrobium nobile*	Netherlands	MG600738	MG600800	–	–	MG600944	MG601005	**–**	**–**
* C. piperis*	IMI 71397, CPC 21195^a^	*Piper nigrum*	Malaysia	MG600760	MG600820	–	–	MG600964	MG601027		
*C. plurivorum*	CBS 125474^a^	*Coffea* sp.	Vietnam	MG600718	MG600781	–	–	MG600925	MG600985	**–**	**–**
	CBS 132443	*Coffea sp.*	Vietnam	MG600717	MG600780	–	–	MG600924	MG600984	**–**	**–**
	CMM 3742	*Mangifera indica*	Brazil	KC702980	KC702941	–	–	KC702908	KC992327	**–**	**–**
	CMM 3746	*Mangifera indica*	Brazil	KC702981	KC702942	–	–	KC702909	KC992328	**–**	**–**
	CORCG2	*Cymbidium* *hookerianum*	China	HM585397	HM585380	–	–	HM581985	HM585422	**–**	**–**
	**CPC 28638**	***Capsicum annuum***, **leaf**	**Thailand**	**MH805810**	**MH805816**	–	–	**MH805828**	**MH805824**	**–**	**–**
	**CPC 28639**	***Capsicum annuum***, **leaf**	**Thailand**	**MH805811**	**MH805817**	–	–	**MH805829**	**MH805825**	**–**	**–**
	LJTJ 16	*Capsicum annuum*	China	KP748207	KP823786	–	–	KP823739	KP823851	**–**	**–**
	LJTJ 22	*Capsicum annuum*	China	KP748213	KP823792	–	–	KP823740	KP823852	**–**	**–**
	LJTJ 30	*Capsicum annuum*	China	KP748221	KP823800	–	–	KP823741	KP823853	**–**	**–**
	**UOM 1004**	***Capsicum annuum***	**Thailand**	**MH805812**	**MH805818**	–	–	**MH805830**	**MH805824**	**–**	**–**
	**UOM 1005**	***Capsicum annuum***	**Thailand**	**MH805813**	**MH805819**	–	–	**MH805831**	**MH805825**	**–**	**–**
	**UOM 1006**	***Capsicum annuum***	**Thailand**	**MH805814**	**MH805820**	–	–	**MH805832**	**MH805826**	**–**	**–**
	**UOM 1153, M2**	***Capsicum annuum***	**Malayasia**	**MH805815**	**MH805821**	–	–	**–**	**MH805827**	**–**	**–**
*C. sojae*	CAUOS5	*Capsicum *sp.	China	KP890107	KP890138	–	–	–	KP890114	**–**	**–**
	ATCC 62257^a^	*Glycine max*	USA	MG600749	MG600810	–	–	MG600954	MG601016	**–**	**–**
*C. vittalense*	CBS 181.82^a^	*Theobroma cacao*	India	MG600734	MG600796	–	–	MG600940	MG601001	–	–

*1ATCC* American Type Culture Collection, *BBA* Culture collection of the Biologische Bundesanstalt fur Land- und Forstwirtschaft, Berlin, Germany, *BRIP* Queensland Plant Pathology Herbarium, Australia, *CPC* Culture collection of P.W. Crous, housed at Westerdijk Fungal Biodiversity Institute, *CBS* Westerdijk Fungal Biodiversity Institute, Utrecht, The Netherlands, *CGMCC* China, General Microbiological Culture Collection, Beijing, China, *DPI* Department of Primary Industries, *HKUCC* The University of Hong Kong Culture Collection, Hong Kong, China, *ICMP* International Collection of Microorganisms from Plants, Landcare Research, Auckland, New Zealand, *IMI* Culture collection of CABI Europe UK Centre, Egham, UK, *LC* Working collection of Lei Cai, housed at CAS, China, *LF* Working collection of Fang Liu, housed at CAS, China, *MFLUCC* Mae Fah Luang University Culture Collection, ChiangRai, Thailand, *NBRC* NITE Biological Resource Center, Chiba, Japan, *PD* Plantenziektenkundige Dienst Wageningen, Netherlands, *UOM* University of Melbourne culture collection, Victoria, Australia, *ZJUD* Diaporthe strains in Zhejiang University, China. Cultures indicated with an asterisk (^a^) are ex-type cultures

Isolates and accession numbers in bold represents the isolates used in this study

### Phylogenetic analyses

Gene sequences of each isolate were examined using Geneious Pro v. 11.1.4, aligned by CLUSTALW2 (Larkin et al. 2007) and edited manually where necessary. ITS and *tub2* sequences of selected isolates representing all the species complexes were analysed to determine to which clade each isolate belonged, and an initial phylogenetic tree was produced with a maximum likelihood analysis (ML) as implemented in MEGA v. 6 with 1000 bootstrap replicates (data not shown). For isolates from the acutatum complex, concatenated datasets were generated comprising ITS, *chs-1, act, gapdh, his3* and *tub2* gene sequences. For isolates from the gloeosporioides complex, two concatenated datasets were generated comprising ITS, *chs-1, act, gapdh* and *tub2* gene sequences, and comprising *ApMat* and *gs* gene sequences. For isolates from the boninense and orchidearum complexes concatenated datasets were generated comprising ITS, *gapdh, act* and *tub2* gene sequences. Selected reference or ex-type strains from each complex (Table 2) were included in the analyses (Damm et al. 2012b, 2019; Marin-Felix et al. 2017; Weir et al. 2012).

Further phylogenetic analyses were performed using MrBayes v. 3.2.6 (Ronquist et al. 2012) for Bayesian inference analyses (BI), and PAUP (Phylogenetic Analysis Using Parsimony) v. 4.0b10 (Swofford 2003) for parsimony analyses. For BI analyses, the best nucleotide substitution model for each locus was determined by MrModeltest v. 2.3 (Nylander 2004) (Table 3), and eight simultaneous MCMC chains were run for 1 bn generations. Trees were sampled every 100 generations for the acutatum, boninense and orchidearum complexes, and every 1000 generations for the gloeosporioides complex 2-gene alignment and every 10 generations for the gloeosporioides complex 5-gene alignment. The heating parameter was set to 0.2 and analyses stopped once the average standard deviation of split frequencies was below 0.01. The first 25% of trees, representing the burn-in phase of the analyses, were discarded and the remaining trees in each analysis were used to calculate posterior probabilities. The generated 50% majority rule consensus tree was viewed in TreeView v. 1.6.6 (Page 1996). A maximum parsimony (MP) analysis was performed on the multilocus alignments as well as for each gene separately with PAUP v. 4.0b10 (Swofford 2003) using the heuristic search option with 100 random sequence additions and tree bisection and reconstruction (TBR) as the branch-swapping algorithm. Gaps were treated as new character states and missing data as missing characters. Bootstrap support values were calculated based on 1000 bootstrap replicates. Statistical measures calculated included tree length (TL), consistency index (CI), retention index (RI) and rescaled consistency index (RC) (Table 3). Alignments and tree files are deposited in TreeBASE (accession https://www.treebase.org/treebase-web/home.html; study S23829).

**Table 3:** Statistical information of the different phylogenetic analyses performed on each *Colletotrichum* complex

Dataset	Parameters and statistics of the Bayesian analyses									Total number of generations run
	Substitution models (Number of Unique site patterns)								Number of trees used in consensus	
	ITS	*gapdh*	*tub2*	*act*	*chs-1*	*his3*	*ApMat*	*gs*		
acutatum complex	HKY + I (108)	SYM + G (151)	GTR + G (134)	GTR + G (86)	K80 + I (54)	GTR + G (96)			45,602	3,040,000
boninense complex	HKY + I (42)	HKY (130)	HKY + G (111)	HKY + G (94)	HKY + G (55)				12,002	80,000
gloeosporioides complex, 2-gene							HKY + G (520)	GTR + G (432)	442,502	29,500,000
gloeosporioides complex, 5-gene	SYM + I (73)	HKY + G (163)	SYM + I (180)	HKY + I (85)	K80 + G (55)				102,752	685,000
orchidearum complex	GTR + I (34)	HKY (55)	HKY (90)	HKY (42)					4128	275,000
	Statistics of the parsimony analyses									
	Number of strains (incl. Outgroup(s))	Number of included characters	Number of parsimony-informative characters	Number of parsimony-uninformative characters	Number of constant characters	Tree Length (TL)	Consistency index (CI)	Retention index (RI)	Rescaled consistency index (RC)	Number of equally most parsimonious trees saved
acutatum complex	100	2210	282	438	1490	1190	0.76	0.79	0.6	1000
boninense complex	24	1743	189	343	1211	776	0.87	0.79	0.68	3
gloeosporioides complex, 2-gene	92	1715	559	539	617	2003	0.73	0.88	0.64	161
gloeosporioides complex, 5-gene	85	1724	306	222	1196	926	0.71	0.857	0.610	1000
orchidearum complex	26	1417	72	282	1063	411	0.92	0.85	0.78	284

### Pathogenicity assay

Pathogenicity tests on chili fruit were conducted using only *Colletotrichum* isolates with straight conidia as previous studies had extensively studied the pathogenicity of *C. truncatum* in chili (Mongkolporn et al. 2010, Ranathunge et al. 2012). There were 15 representative isolates of *C. scovillei* from Indonesia, Thailand and Taiwan, 10 isolates of *C. siamense* from Indonesia and Thailand, and one isolate each from the other eight species with straight conidia. Detached mature red chili fruits (*Capsicum annuum* genotype Bangchang) were used for the pathogenicity assay as described by De Silva et al. (2017a). Pathogenicity of each isolate was tested with both non-wound and wound inoculation methods. Three replicate fruits were tested per isolate while experiments were carried out three times.

Data were analysed using the Mixed Procedure in SAS v. 9.4 by fitting the linear mixed model:

*Y*_*ijkl*_ = *μ* + *S*_*i*_ + *I*_*j*_(*S*_*i*_) + *R*_*k*_ + *R*_*k*_ ∗ *S*_*i*_ + *R*_*k*_ ∗ *I*_*j*_(*S*_*i*_) + *e*_*ijkl*_

where *μ* is the grand mean, *S*_*i*_ is the fixed species effect, and *R*_*k*_, *R*_k_*S_*i*_, *R*_k_*I_*j*_*(S*_*i*_*)* and *e*_*ijkl*_ are respectively the random effects of replicate, replicate by species interaction, replicate by isolate within species interaction, and error. Separate analyses were done for wound and non-wound data as preliminary analysis showed there was significant species by wound interaction. Least squared means were estimated for each species and t-test carried out between each pair of means.

## RESULTS

### Isolates

The *Colletotrichum* isolates with falcate conidia and ITS sequences matching to those of the ex-type of *C. truncatum* were the most common (*n* = 115), representing 44% of all isolates. *Colletotrichum truncatum* was found in the collections from Indonesia, Malaysia, Sri Lanka and Thailand (Fig. 6). *Colletotrichum truncatum* isolates were not included in the collection from the World Vegetable Center in Taiwan as only the species with straight conidia were selected for identification. The remaining 56% of isolates (*n* = 145) were of species with straight conidia that mostly belonged to the acutatum and gloeosporioides complexes.

### Phylogenetic analyses

#### Acutatum complex

**Fig. 1:**
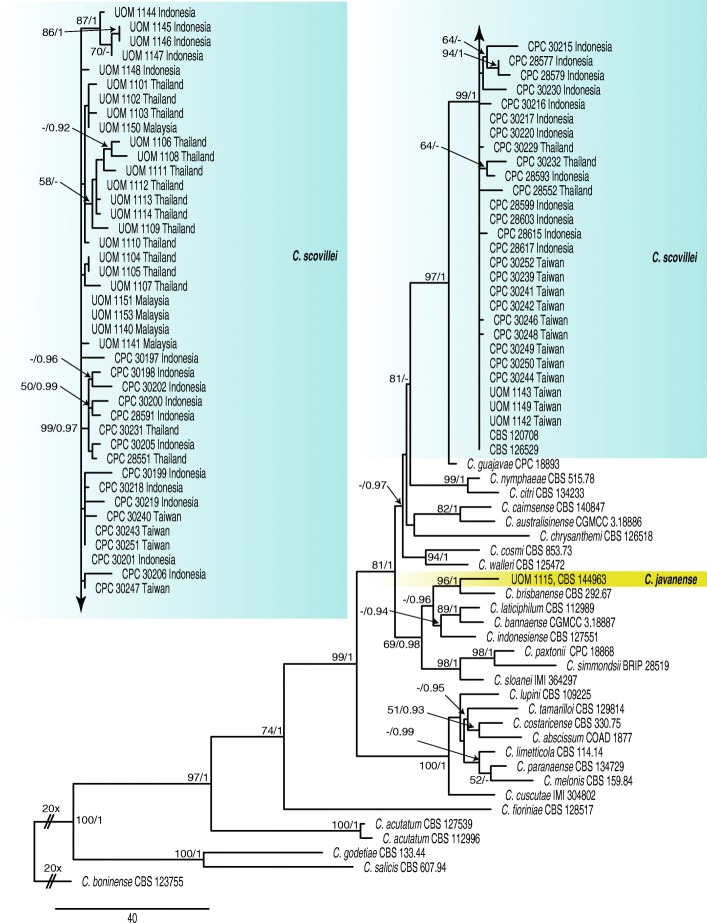
First of 1000 equally most parsimonious trees obtained from a heuristic search of the combined ITS*, tub2, gapdh, chs-1, his3* and *act* sequence alignment of the *Colletotrichum* isolates in the acutatum complex. The parsimony bootstrap support values (MP > 49%) and Bayesian posterior probabilities (PP > 0.90) are displayed at the nodes (MP/PP). The tree was rooted to *C. boninense* (CBS 123755). The bar indicates 40 changes. Coloured blocks are used to indicate clades containing isolates from chili

For the 69 isolates and 29 reference species in the acutatum complex, the phylogenetic analysis of the combined data sets using six genes (ITS, *tub2, gapdh, chs-1, act* and *his3*) with *C. boninense* (CBS 123755) as the outgroup comprised 100 isolates including the outgroup and 2315 characters including the alignment gaps and excluded characters. The Bayesian analysis of this alignment, based on 629 unique site patterns (ITS: 108, *tub2*: 134, *gapdh*: 151, *act*: 86, *chs-1*: 54 and *his3*: 96) lasted 3,040,000 generations, resulting in 60,802 total trees of which 45,602 trees were used to calculate the posterior probabilities. The parsimony analysis yielded the maximum of 1000 equally most parsimonious trees. Bootstrap support values of the MP analysis (MP > 49%) and the BI posterior probabilities (PP > 0.90) were plotted at the nodes (Fig. 1). Overall, the species clades recognised received similar support values, although the association between species did not always receive similar support, e.g. the node linking *C. paranaense* and *C. melonis* (MP < 50% / PP = 0.99). The phylogenetic analyses of the acutatum complex identified *C. scovillei* as the most prevalent species in Indonesia, Malaysia, Thailand and Taiwan*.* However, *C. scovillei* was not isolated from Sri Lanka. In addition, an isolate from Java in Indonesia (UOM 1115) clustered related to *C. brisbanense* (96% BS/1 PP; Fig. 1).

#### Boninense complex

**Fig. 2:**
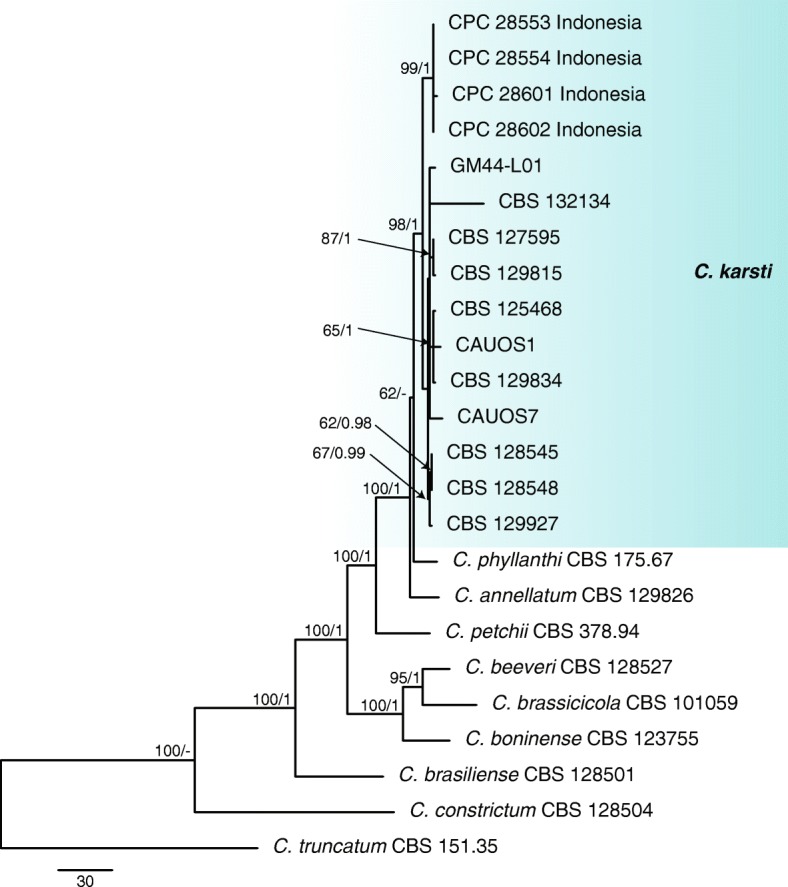
First of three equally most parsimonious trees obtained from a heuristic search of the combined ITS, *tub2*, *gapdh*, *chs-1* sequence alignment of the *Colletotrichum* isolates in the boninense complex. The parsimony bootstrap support values (MP > 49%) and Bayesian posterior probabilities (PP > 0.90) are displayed at the nodes (MP/PP). The tree was rooted to *C. truncatum* (CBS 151.35). The bar indicates 30 changes. Coloured blocks are used to indicate clades containing isolates from chili

For the four isolates and 10 reference species in the boninense complex the phylogenetic analyses of the combined data sets using five genes (ITS, *gapdh, tub2, act* and *chs-1*) with *C. truncatum* (CBS 151.35) as the outgroup comprised 24 isolates and 1867 characters including the alignment gaps and excluded characters (Fig. 2). The Bayesian analysis of the combined alignment, based on 432 unique site patterns (ITS: 42, *gapdh*: 130*, tub2*: 111*, act*: 94 and *chs-1*: 55) lasted 80,000 generations, resulting in 16,002 total trees of which 12,002 trees were used to calculate the posterior probabilities. The parsimony analysis yielded three equally most parsimonious trees. Bootstrap support values of the MP analysis (MP > 49%) and the BI posterior probabilities (PP > 0.90) were plotted at the nodes (Fig. 2). Overall, the nodes received similar support values, except for the subclustering of strains CBS 128545, CBS 128548 and CBS 129927 in the *C. karsti* clade (MP 67% / PP = 0.98).The phylogenetic analyses of the boninense complex identified the most prevalent species as *C. karsti* occurring only in Indonesia.

#### Gloeosporioides complex

**Fig. 3:**
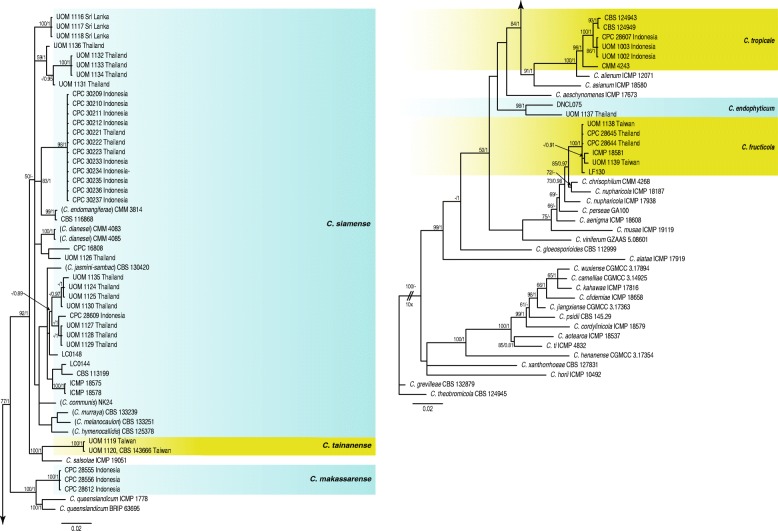
Phylogenetic analysis of *Colletotrichum* isolates in the gloeosporioides complex based on a 50% majority rule consensus tree derived from Bayesian analysis of the *ApMat* and *gs* regions. The parsimony bootstrap support values (MP > 49%) and Bayesian posterior probabilities (PP > 0.90) are displayed at the nodes (MP/PP). The tree was rooted to *C. theobromicola* (CBS 124945). The bar indicates 0.02 expected changes per site. Coloured blocks are used to indicate clades containing isolates from chili

**Fig. 4:**
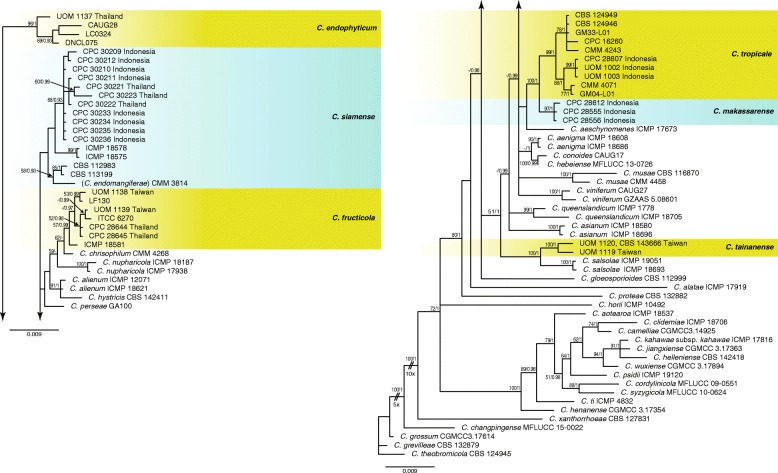
Phylogenetic analysis of *Colletotrichum* isolates in the gloeosporioides complex based on a 50% majority rule consensus tree derived from Bayesian analysis of the combined the ITS, *tub2*, *gapdh*, *chs-1* and *act* sequence. The parsimony bootstrap support values (MP > 49%) and Bayesian posterior probabilities (PP > 0.90) are displayed at the nodes (MP/PP). The tree was rooted to *C. theobromicola* (CBS 124945). The scale bar indicates 0.009 expected changes per site. Coloured blocks are used to indicate clades containing isolates from chili

For the 42 isolates and the 41 reference species in the gloeosporioides complex, two phylogenetic trees were constructed, one from the *ApMat* and *gs* sequence alignment and the second from the *ITS, gapdh, act, tub2, chs-1* sequence alignment (Figs. 3 and 4). The analyses using the 5-gene alignment with *C. theobromicola* CBS 124945 as the outgroup (Fig. 4) comprised 85 isolates including the outgroup and 1863 characters including the alignment gaps and excluded characters. The Bayesian analysis of the combined alignment, based on 556 unique site patterns (ITS: 73, *gapdh*: 163, *act*: 85, *tub2*: 180, *chs-1*: 55) lasted 685,000 generations, resulting in 137,002 total trees of which 102,752 trees were used to calculate the posterior probabilities. The parsimony analysis yielded the maximum of 1000 equally most parsimonious trees. Bootstrap support values of the MP analysis (MP > 49%) and the BI posterior probabilities (PP > 0.90) were plotted at the nodes (Fig. 4). Overall, the species clades recognised in this study received similar support values, except for the *C. siamense* clade (MP < 50% / PP < 0.91) and the *C. fructicola* clade (MP 57% / PP = 0.99).

The analysis using the *ApMat* and *gs* sequence alignment comprised of 92 isolates with *C. theobromicola* CBS 124945 as the outgroup (Fig. 3) and 1824 characters including the alignment gaps and excluded characters. The Bayesian analysis of the combined alignment, based on 952 unique site patterns (*ApMat*: 520, *gs*: 432) lasted 29,500,000 generations, resulting in 590,002 total trees, of which 442,502 trees were used to calculate the posterior probabilities. The parsimony analysis yielded 161 equally most parsimonious trees. Bootstrap support values of the MP analysis (MP > 49%) and the BI posterior probabilities (PP > 0.90) were plotted at the nodes (Fig. 3). Overall, the species clades recognised in this study received similar support values, except for the *C. siamense* clade (MP = 92% / PP < 0.91).

Phylogenetic analyses of the gloeosporioides species complex identified 69% (29) of the chili fruit isolates as *C. siamense*. In the 2-gene tree a distinct subclade within the *C. siamense* clade formed with 100% bootstrap support which contained isolates from Chiang Mai in Thailand, and Gowa and Jeneponto in South Sulawesi of Indonesia (Fig. 3). These isolates from Indonesia and Thailand had very distinct sequences compared to the ex-type reference *C. siamense* strain (CBS 130417) with 28 bp difference in the *gs* gene and 25 bp difference in the *ApMat* gene. A significant sub-clade formed within *C. siamense* with full (100% BS/1 PP) support values in the 2-gene tree. The same isolates in the 5-gene tree did not show the same level of difference but showed a strong similarity between the *C. siamense* isolates. In the 2-gene tree there were also significant subclades of isolates associated with different geographical regions, in particular the distinct subclade of the Sri Lankan isolates (UOM 1116, UOM 1117, UOM 1118) from Kandy and the separate subclade of Thai isolates from Ratchaburi (UOM 1132, UOM 1133, UOM 1134).

Other species identified in the gloeosporioides complex included *C. endophyticum* and *C. fructicola* from Thailand, *C. fructicola* and *C. tainanense* sp. nov. from Taiwan, and *C. tropicale* and *C. makassarense* sp. nov*.* from Indonesia. Most of the identified species including the two new species were supported in distinct clades with significant bootstrap values in both the 5-gene and 2-gene trees (Figs. 3 and 4). However, due to a lack of sequence data of the *ApMat* gene for some reference strains, it was difficult to provide a good support for placement of some species such as *C. endophyticum* in the 2-gene trees. Three isolates (CPC 28607, UOM 1002, UOM 1003) collected from the Makassar region in Indonesia showed a close relationship to the reference species *C. tropicale* in the *ApMat* and *gs* tree (Fig. 3). Individual gene trees of ITS, *act, tub2, chs-1* loci (data not shown) also supported these isolates as *C. tropicale*. Nevertheless, in the 5-gene tree a separate sub clade was formed with full support (100% BS/1 PP) different to the *C. tropicale* reference species (Fig. 4). In both trees, two isolates (UOM 1120, UOM 1119) collected from Tainan in Taiwan formed a significant distinct clade with full support (100% BS/1 PP) separate from *C. salsolae.*

#### Orchidearum complex

**Fig. 5:**
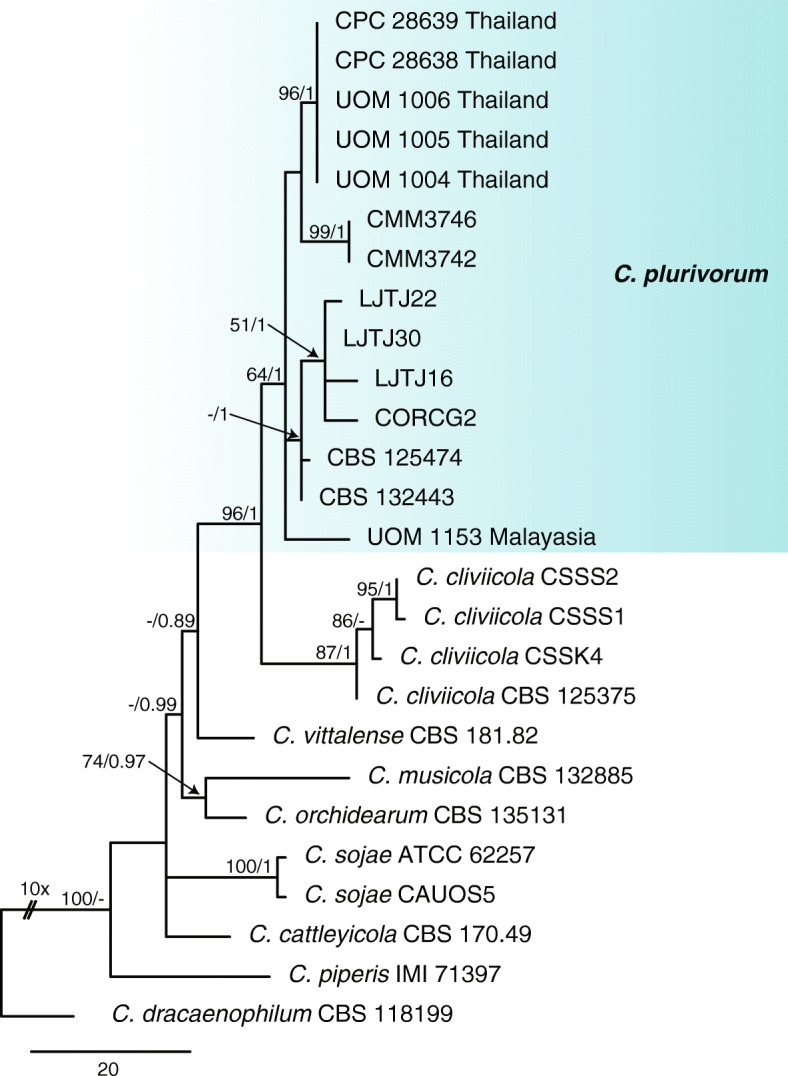
First of 284 equally most parsimonious trees obtained from a heuristic search of the combined ITS, *tub2*, *gapdh*, *chs-1*, and *act* sequence alignment of the *Colletotrichum* isolates in the orchidearum complex. The parsimony bootstrap support values (MP > 49%) and Bayesian posterior probabilities (PP > 0.90) are displayed at the nodes (MP/PP). The tree was rooted to *C. dracaenophilum* (CBS 118199). The scale bar indicates 20 changes. Coloured blocks are used to indicate clades containing isolates from chili

For the six isolates and nine reference species in the orchidearum complex the phylogenetic analysis of the combined data sets using four genes (ITS, *gapdh, tub2* and *act*) with *C dracaenophilum* (CBS 118199) as the outgroup comprised 26 isolates and 1543 characters including the alignment gaps and excluded characters. The Bayesian analysis of the combined alignment, based on 221 unique site patterns (ITS: 34, *gapdh*: 55, *act*: 42, *tub2*: 90) lasted 275,000 generations, resulting in 5502 total trees of which 4128 trees were used to calculate the posterior probabilities. The parsimony analysis yielded 284 equally most parsimonious trees. Bootstrap support values of the MP analysis (MP > 49%) and the BI posterior probabilities (PP > 0.90) were plotted at the nodes (Fig. 5). Overall, the species clades recognised in this study received similar support values, except for the *C. plurivorum* clade (MP = 64% / PP <  1) and the *C. cliviicola* clade (MP = 87% / PP <  1).

The phylogenetic analyses identified the isolates from Thailand and Malaysia as *C. plurivorum*. Five isolates collected from Thailand, including three taken from infected chili leaves from Chiang Rai and another two isolates collected from infected green chili fruit from Bangkok, formed a poorly supported subclade within *C. plurivorum* (Fig. 5).

## TAXONOMY

Morphological observations and phylogenetic data of the straight conidia species clearly identified three novel species, two from Indonesia and one from Taiwan. Detailed morphological descriptions are provided below for all the *Colletotrichum* species associated with chili anthracnose (Table 4).

**Table 4:** Morphological characteristics of *Colletotrichum* species causing anthracnose of chili

Species	Conidiogenous cells length (μm)	Conidia length (μm)	Conidia width (μm)	Appressoria (μm)
*C. endophyticum*	12–21 × 3–4	(10.4–)12.5–13(−14.5)	(3–)4.5–5(−6.3)	(10.5–)12(−15) × (3–)4.5(−10)
*C. fructicola*	7–17.5	(10.5–)12.5–13(−18.5)	(3–) 4–5.5(−6.5)	–
*C. javanense*	7–17.5	(11.5–)13.5–14(−15.8)	(2.4–)3.5–4(−4.3)	(6–)8.2(−11.3) × (4.2–)5.6(−7.5)
*C. karsti*	–	(11.6–)12.5–13(−15.7)	(3–)4–5.2(−6.5)	6–12.5 × 3.5–8.2
*C. makassarense*	7–25 × 3–4	(11–)13–15(−17)	(4–)4.5–5	(6–)8(−10.5) × (4–)3.5(−8.6)
*C. plurivorum*	26–48 × 3–4	(13.7–)14–16(− 18.3)	(3.8–)5(−5.6)	(10.5–)12(−23) × (3.5–)5.5(− 11.5)
*C. scovillei*	7–17.5	(5.5–)9.5–10(−12)	(2.4–)3(−3.8)	(4–)5.5(−12.5) × (3.5–)4.5–5(− 6.5)
*C. siamense*	6.5–16	(13–)14(− 15.5)	(3–)4.2(−5.3)	(4.5–)7.5(− 10) × (3.5–)3(−5.5)
*C. tainanense*	–	(16–)17–18(−22)	(4.5–)5	(6.5–)10.3(− 14.3) × (6.2–)5.2(−9.5)
*C. tropicale*	7–15 × 3.5–4.5	(13–)14–16(− 17)	(3.5–)4–5(− 6)	–

### *Colletotrichum javanense* D.D. De Silva, P.W. Crous & P.W.J. Taylor, sp. nov. MycoBank MB826936.

**Fig. 6:**
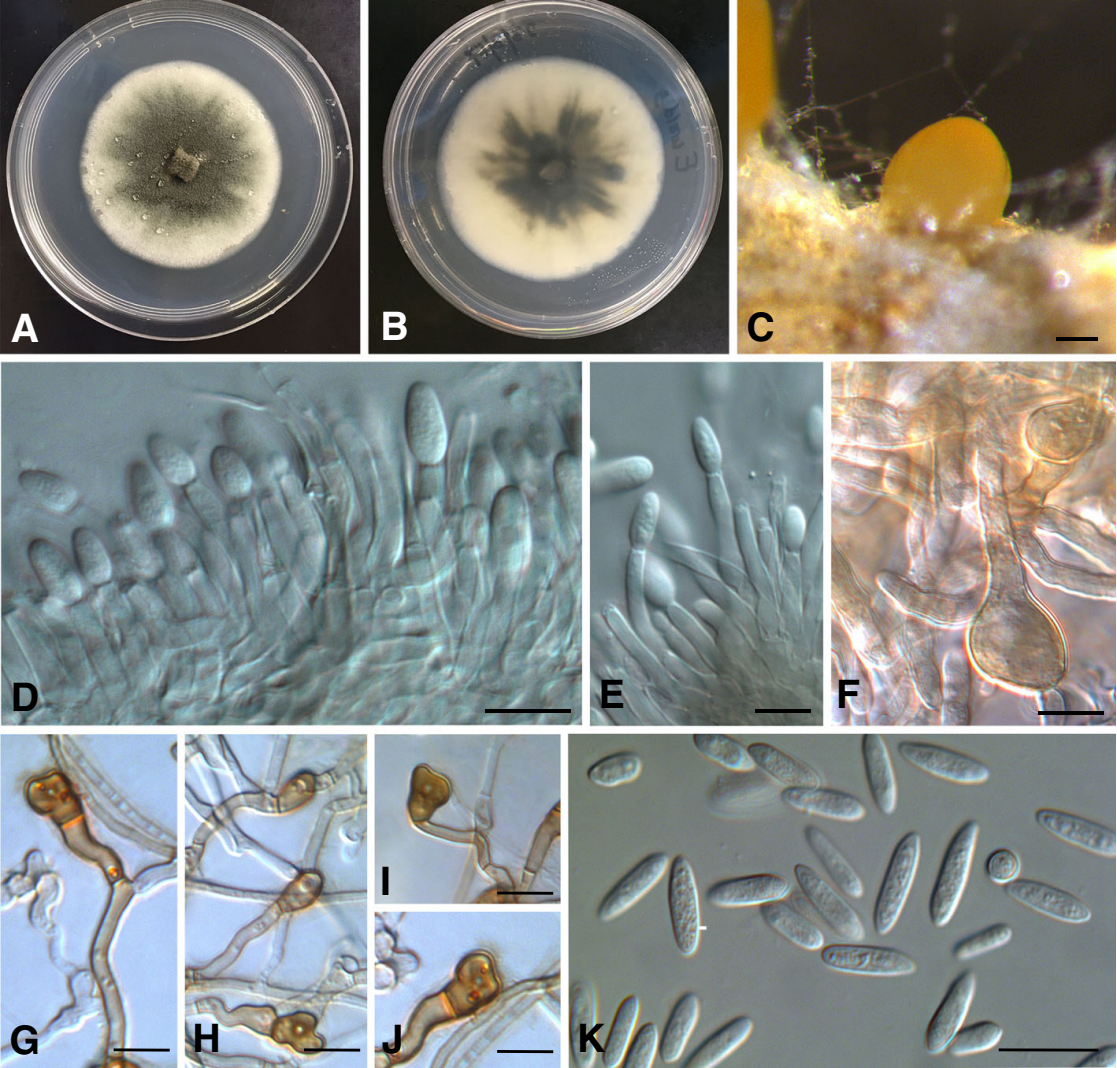
*Colletotrichum javanense* (CBS 144963). **a** Colony on PDA. **b** Reverse side of the colony on PDA. **c** Conidioma on PDA. **d-e** Conidiophores and conidia. **f** Chlamydospores*.* **g–j** Appressoria. **k** Conidia. Bars = 10 μm

Figure 6 *Etymology*: Named after Java, the island in Indonesia where the species was collected.

*Diagnosis*: *Colletotrichum javanense* differs from *C. brisbanense* in forming distinct chlamydospores and acervular conidiomata on all the media tested*.* In contrast, *C. brisbanense* only produced *Conidiomata* on *Anthriscus stem*, but no basal cells observed. In addition, *C. javanense* grows faster than *C. brisbanense* (*C. javanense* on OA, MEA and SNA 60, 55 and 66 mm diam in 7 d, respectively, *C. brisbanense* on OA and SNA 18.5, 20 mm diam in 7 d, respectively). *Colletotrichum javanense* is phylogenetically distinct to *C. brisbanense* with both species being different in the sequences of *chs-1*, and most effectively with *gapdh* (7 bp difference) and *his3* (4 bp difference).

*Type*: **Indonesia**: West Java, Purwakata regency, on fruit of *Capsicum annuum*, Dec. 2014, *F. Fitriyah* (CBS H-144963 – holotype; CBS 144963 = UOM 1115 = EWINDO 3 – ex-type cultures).

*Description*: *Sexual morph* not observed. *Asexual morph* on PDA. *Vegetative mycelium* 1–5 μm diam, hyaline, smooth-walled, septate, branched hyphae. *Chlamydospores* globose or elongate, pale brown, smooth-walled, 5–25 × 3–8 μm. *Conidiomata* acervular, *setae* not observed. *Conidiophores* hyaline, septate, branched. *Conidiogenous cells* hyaline, cylindrical or ampulliform, 7–17.5 μm, apex 1–3 μm diam. *Conidia* hyaline, aseptate, smooth-walled, mostly fusiform, one end rounded, the other end acute, or both ends acute (11.5–)13.5–14(− 16) × (2.5–) 4(− 4.5) μm. Conidia in mass yellow to orange colour. *Appressoria* single or in loose groups, medium brown, smooth-walled, subglobose or elliptical, with entire or undulate margin, (6–)8(− 11.5) × (4–)6(− 7.5) μm.

*Asexual morph* on SNA. *Vegetative mycelium* 1–7 μm diam, hyaline, smooth-walled, septate, branched hyphae. *Chlamydospores* globose or elongate, pale brown, smooth-walled, 4.5–28 × 4–8 μm. *Conidiomata* acervular, *setae* not observed. *Conidiophores* hyaline, septate, branched, 20–35 μm long. *Conidiogenous cells* hyaline, cylindrical or ampulliform, 5–20 μm, apex, 1–3 μm diam. *Conidia* hyaline, aseptate, smooth-walled, cylindrical with both ends acute or one end round and one end acute, (13.5–)16.5(− 24) × (2.5–) 3(− 4.5) μm. Conidia in mass with yellow to orange colour.

*Culture characteristics: Colonies* on PDA 48–54 mm diam in 7 d (6.5–7.5 mm/d), flat with entire margin; surface covered with grey to olive-green short aerial mycelium, margin white to light grey, reverse mostly cream whitish, olivaceous grey to black in the centre. Yellow to orange acervular conidiomata*.* Colonies on SNA were 60–66 mm diam in 7 d (8–9.5 mm/d), flat with entire margin, hyaline to pale brown, surface covered with short grey aerial mycelium, reverse same colours. Orange acervular conidiomata at the centre of the culture. Colonies on OA were 55–60 mm diam in 7 d (7.8–8.5 mm/d), flat with entire margin; surface covered with cream to grey short aerial mycelium, margin white, reverse mostly light orange, with brown pigments. Orange acervular conidiomata*.* Colonies on MEA surface pale grey short aerial mycelium, reverse light orange.

*Notes*: The closest match in a blastn search with the *gapdh* sequence was GenBank JQ948617, *C. sloanei* strain IMI 364297 with 98% identity (4 bp differences), while the closest matches with the *his3* sequence with 99% identity (2 bp differences) were GenBank JQ949279 *C. indonesiense* strain CBS 127551 and GenBank KJ947248 *C. guajavae* isolate OBP19.

### *Colletotrichum makassarense* D.D. De Silva, P.W. Crous & P.W.J. Taylor, sp. nov. MycoBank MB827691.

**Fig. 7:**
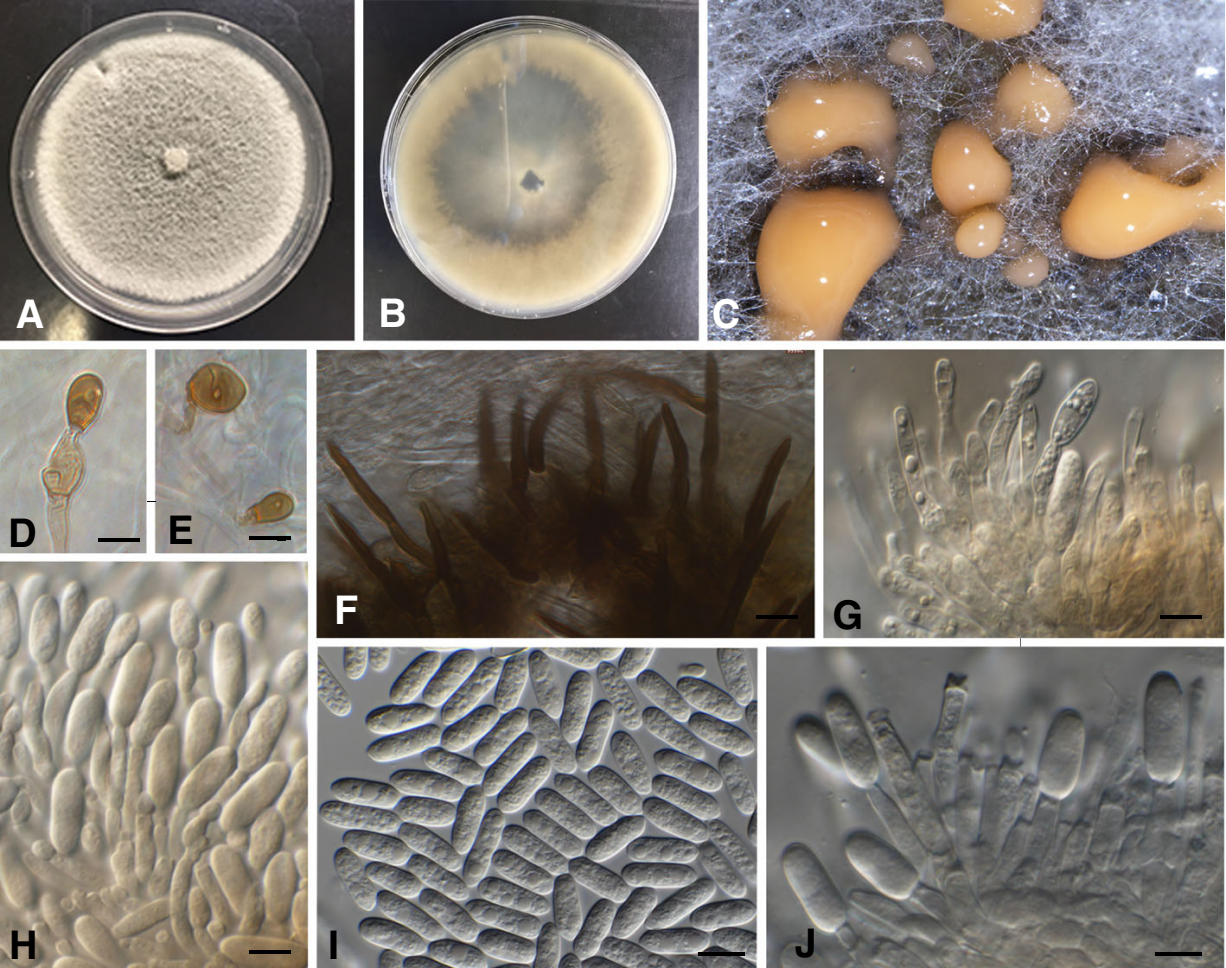
*Colletotrichum makassarense* (CBS 143664)*.* **a** Colony on PDA. **b** Reverse side of the colony on PDA. **c** Conidiomata. **d–e** Appressoria. **f** Setae, **g, h, j** Conidiophores and conidia. **i** Conidia. Bars = 10 μm

Figure 7 *Etymology*: Named after Makassar, the city in South Sulawesi, Indonesia, where the species was collected.

*Diagnosis*: *Colletotrichum makassarense* is phylogenetically closely related to *C. tropicale*. Sequence data from ITS could not separate the two species, but they can be distinguished by all other genes tested and most effectively using *ApMat* (22 bp differences) and *gs* (18 bp differences) sequence data.

*Type*: **Indonesia**: Makassar, from fruit lesion of *Capsicum annuum*, 7 Jun. 2015, *P.W.J. Taylor & A. Nasruddin* (CBS H-143664 – holotype; CBS 143664 = CPC 28612 – ex-type cultures).

*Description*: *Sexual morph* not observed. *Asexual morph* on OA. *Vegetative mycelium* consisting of hyaline, smooth-walled, septate, branched hyphae, 2–3 μm diam. *Chlamydospores* not observed. *Setae* present, medium brown, 40–55 × 3–5 μm, 2–3-septate, tapering to acute apices. *Conidiomata* acervular, 100–200 μm diam, with orange conidial masses. *Conidiophores* subcylindrical, flexuous, 1–4-septate, hyaline, smooth, branched, 15–45 × 3–4 μm. *Conidiogenous cells* subcylindrical, hyaline, smooth, phialidic with periclinal thickening, 7–25 × 3–4 μm. *Conidia* hyaline, smooth, aseptate, subcylindrical, straight, apex obtuse, tapering at base to protruding truncate hilum, 1 μm diam, prominently guttulate, (11–)13–15(− 17) × (4–)4.5–5 μm. *Appressoria* solitary, medium brown, smooth-walled, subglobose, ellipsoidal to obovate, entire margin, (6–)8.0(− 10.5) × (4–)3.5(− 8.5) μm.

*Culture characteristics*: *Colonies* on PDA 45 mm diam after 7 d (6.5 mm/d), colonies flat, with moderate aerial mycelium, on OA surface smoke-grey. On PDA surface smoke-grey, reverse olivaceous grey. On MEA surface dirty white, reverse ochreous.

*Notes*: The closest match in a blastn search with the *ApMat* sequence was GenBank KU923732, *C. queenslandicum* strain AUS22 with a 98% identity (16 bp differences), while the closest match with the *gs* sequence with 99% identity (7 bp differences) was GenBank KJ947286 *C. siamense* isolate OBP24. The best matches with the *gapdh* sequence were GenBank KX578784 *C. siamense* (99% identity, 3 bp differences) and GenBank KU221347 *C. queenslandicum* (99%, identity, 3 bp differences).

### *Colletotrichum tainanense* D.D. De Silva, P.W. Crous & P.W.J. Taylor, sp. nov. MycoBank MB827692.

**Fig. 8:**
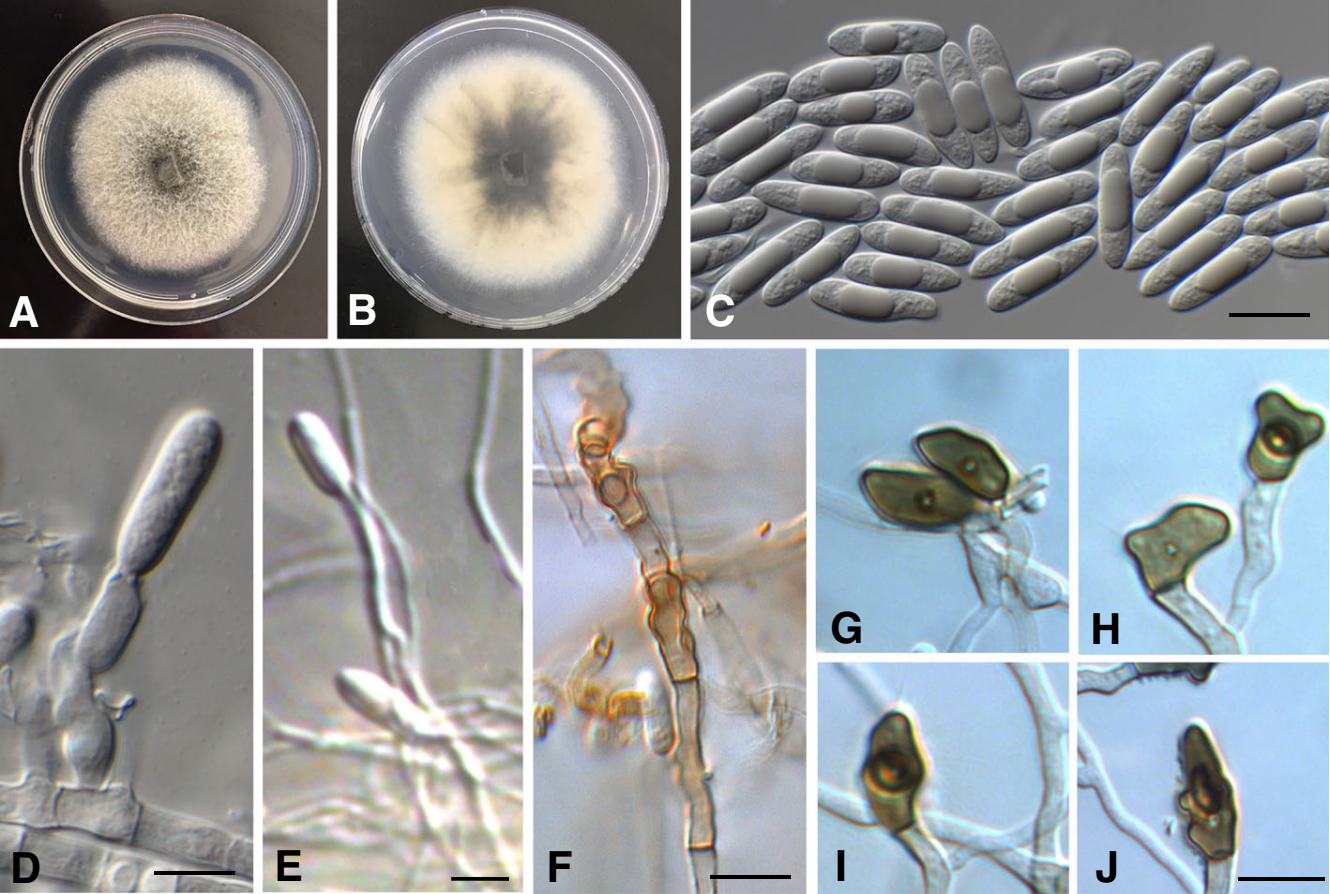
*Colletotrichum tainanense* (CBS 143666). **a** Colony on PDA. **b** Reverse side of the colony on PDA. **c** Conidia. **d-e** Conidiophores and Conidia. **f** Melanised hypae **g-j** Appressoria. Bars = 10 μm

Figure 8 *Etymology*: Named after Tainan, the city in Taiwan where the species was collected.

*Diagnosis*: *Colletotrichum tainanense* differs from its closest phylogenetic neighbour *C. salsolae* in the culture characteristics on PDA, sparse aerial mycelium and pale mouse-grey surface mycelium, whereas *C. salsolae* produces a layer of acervuli-like structures with deep orange brown conidial masses and numerous setae. The two species are separable using all the genes tested except for ITS and most effectively with *gapdh* (7 bp difference), *tub2* (6 bp difference) and *act* (5 bp difference) sequences. There is only one bp difference in the *chs-1* sequence between the two species.

*Typ*e: **Taiwan**: Tainan: on fruit of *Capsicum annuum*, Aug. 2014, *Z.M. Sheu* (CBS H-143666 – holotype; CBS 143666 = CPC 30245 = UOM 1120 = Coll 1298 – ex-type cultures).

*Description*: *Sexual morph* not observed. *Asexual morph* on PDA. *Vegetative mycelium* branched, hyaline, smooth-walled, septate, hyphae 2–3 μm diam, melanised with time. A single conidioma found on a PDA plate, sterile on SNA, MEA, and OA. *Chlamydospores* and *setae* not observed. *Conidiophores* subcylindrical, flexuous, 1–2-septate, hyaline, smooth to pale brown, branched. *Conidiogenous cells* subcylindrical, hyaline, smooth, phialidic with periclinal thickening. *Conidia* hyaline, smooth, aseptate, subcylindrical to subclavate, straight or slightly curved, apex obtuse, tapering at base to protruding truncate hilum, 1.5–2 μm diam, prominently guttulate, (16–)17–18(− 22) × (4.5–)5 μm. *Appressoria* single or in loose groups, often narrow-cylindric, medium to dark brown, often tapering towards apex, the edge entire or undulate sometimes irregularly lobed (6.5–)10.5(− 14.5) × (6–)5(− 9.5) μm.

*Culture characteristics: Colonies* on PDA 45 mm diam after 7 d (6.5 mm/d), colonies flat, with moderate aerial mycelium. On OA surface pale mouse-grey. On PDA surface pale mouse-grey, reverse mouse-grey. On MEA surface pale mouse-grey, reverse olivaceous grey.

*Notes*: The closest match in a blastn search with the *gapdh* sequence with 99% identity (2 bp difference) was GenBank KC790761 *Colletotrichum* sp. strain MTCC 9664 while the closest match with the *act* sequence with 99% identity (2 bp difference) was GenBank KY995522 *C. siamense* strain LJDY1–2. The closest match with the *tub2* sequence with 99% identity (7 bp difference) was GenBank MF143931 *C. siamense* strain 31-B-1.

### *Colletotrichum endophyticum* Manamgoda et al., *Fung. Diversity* 61: 112 (2013); as *‘endophytica’*.

*Description: Colonies* on PDA 42 mm diam after 7 d (5.5 mm/d), pale orange to white aerial mycelium; reverse pale white to orange and black at the centre and numerous orange conidiomata scattered over the surface. *Chlamydospores* not observed. *Conidiomata* present, conidiophores formed directly on hyphae. Setae present, moderately brown, 47–95 × 3–6 μm, 3–4-septate, tapering acute apices. *Conidiophores* hyaline, smooth-walled and unbranched. *Conidiogenous cells* hyaline, smooth-walled, aseptate, subcylindrical, 12–21 × 3–4 μm. *Conidia* hyaline, smooth-walled, aseptate, straight, cylindrical with two ends obtuse, (10.5–)12.5–13(− 14.5) × (3–)4.5–5(− 6.5) μm. *Appressoria* single or in loose groups, brown, slightly lobed, (10.5–)12(− 15) × (3–)4.5(− 10) μm.

*Notes*: *Colletotrichum endophyticum* was first described as a grass endophyte of *Pennisetum purpureum* from northern Thailand (Manamgoda et al. 2013). Later, it was reported from several other host species including *Capsicum* in China (Diao et al. 2017). The length of conidia of the isolate from Thailand (UOM 1137) was slightly shorter than that of the ex-type (LC0324) of *C. endophyticum* (conidia 13–19(− 21) × (3.5–)4.5–5.5 μm).

*Material examined*: **Thailand**: Kanchanaburi: from fruit lesion of *Capsicum annuum*, 2010, *P.W.J. Taylor & O. Mongkolporn* (culture UOM 1137 = F5-2D).

### *Colletotrichum fructicola* Prihast. et al., *Fung. Diversity* 39: 158 (2009).

*Description: Colonies* on PDA 65 mm diam after 7 d (8.5–11 mm/d), flat with entire edge, aerial mycelium dense, cottony, pale grey to white aerial mycelium and numerous black stroma scattered over the surface, grey in the centre, white at the margin; reverse greyish green. *Chlamydospores* not observed. *Conidiomata* acervular, *Setae* was observed, brown, smooth-walled, 1–2-septate, 60 μm long, tapering acute apices. *Conidiophores* hyaline, septate, branched. *Conidiogenous cells* hyaline, cylindrical or ampulliform, 7–17.5 μm. *Conidia* hyaline, aseptate, smooth-walled, cylindrical, both ends obtuse, (10.5–)12.5–13(− 18.5) × (3–) 4–5.5(− 6.5). *Appressoria* not observed.

*Notes*: The sexual morph of these isolates was not observed in culture. Conidial length of isolate CPC 28644 was slightly longer than that of the ex-type (ICMP 18581 = BPD-I16) of *C. fructicola* (conidia 9.7–14 × 3–4.3 μm, *x* = 11.53 ± 1.03 × 3.55 ± 0.32 μm; Prihastuti et al. 2009).

*Material examined*: **Thailand**: Chiang Mai: from fruit lesion of *Capsicum annuum*, 7 Jun. 2015, *P.W.J. Taylor & O. Mongkolporn* (cultures CPC 28644 and CPC 28645). **Taiwan**: Cyonglin, Hsinchu, from fruit lesion of mature red fruit of *Capsicum* sp. (sweet pepper), 22 Apr. 2015, *Z.M. Sheu* (culture UOM 1139 = coll 1318); Nantou, Renai, from fruit lesion of green fruit of *Capsicum* sp. (sweet pepper), 4 Sep. 2008, *Z.M. Sheu & C. Wang* (culture UOM 1138 = coll-853).

### *Colletotrichum karst*i You L. Yang et al. *Cryptogamie, Mycologie* 32: 241 (2011); as *‘karstii’*.

*Description: Colonies* on PDA 65 mm diam after 7 d (6.5–10.5 mm/d), flat with entire edge, orange to white aerial mycelium and numerous orange conidial masses scattered over the surface, white at the margin; reverse yellow to orange. *Chlamydospores* not observed. *Conidiomata* acervular, setae were observed, brown, smooth-walled, 2–4-septate, 60 μm long, base submerged, tapered towards apex, tip mostly acute. *Conidiophores* hyaline, septate, branched, (10.5–)12–38(− 47.5) × (3–)4–5.5(− 6.5) μm. *Conidiogenous cells* hyaline, cylindrical or ampulliform, 7–15.5 μm. *Conidia* hyaline, aseptate, smooth-walled, short, cylindrical, both ends obtuse or one end slightly acute or truncate at the base, (11.5–)12.5–13(− 15.5) × (3–)4–5(− 6.5) μm. *Appressoria* single or in loose groups, brown, subglobose, circular outline, 6–12.5 × 3.5–8 μm.

*Notes*: The four isolates identified from Indonesia in the boninense complex produced distinct short conidia compared to the ex-epitype culture of *C. karsti* (14.5–17 × 5–6.5 μm; Yang et al. 2011). However, high variability of conidia size between different strains of *C. karsti* were reported by Damm et al. (2012a, 2012b) where the conidia measurements of CBS 129833 were (11.5–)12.5–14(− 14.5) × (5–)5.5–6(− 6.5) μm, mean ± SD = 13.1 ± 0.7 × 5.8 ± 0.4 μm; and CBS 111998 had a conidium length up to 18.5 μm, L/W ratio = 2.8. These isolates did not form a sexual morph in culture. Although these four isolates formed a fully supported (100% BS/1 PP) subclade within *C. karsti*, all the individual gene trees (data not shown) did not provide significant support to justify introducing a cryptic species for these isolates.

*Material examined*: **Indonesia**: Jeneponto, from fruit lesions of *Capsicum annuum*, 7 Jun. 2015, *P.W.J. Taylor & A. Nasruddin* (cultures CPC 28553, CPC 28554); from fruit lesion of mature red fruit and peduncle lesions of *Capsicum* sp. Jun. 2015, *P.W.J. Taylor & A. Nasruddin* (cultures CPC 28601, CPC 28602).

### *Colletotrichum plurivorum* Damm et al., *Stud. Mycol.* 92: 31 (2019).

*Description: Colonies* on PDA 63 mm diam after 7 d (8 mm/d), grey to dark brown aerial mycelium; reverse grey to light brown with yellow-orange in the centre with abundant acervular conidiomata that ooze pale orange conidial masses at the centre, *Chlamydospores* not observed. *Conidiomata* present (near the inoculation point), conidiophores formed directly on hyphae. *Sclerotia* present. *Setae* present, medium brown, 94–125 × 4–6 μm, 1–4-septate, tapering towards acute apices, often with a constriction at the apex. *Conidiophores* hyaline to pale brown, smooth-walled, septate and branched. *Conidiogenous cells* hyaline, smooth-walled, aseptate, subcylindrical, straight to gently curved, 26–48 × 3–4 μm, phialidic, periclinal thickening conspicuous. *Conidia* hyaline, smooth-walled, aseptate, straight, cylindrical with two ends obtuse or one end slightly acute, (13.5–)14–16(− 18.5) × (4–)5(− 6.5) μm. *Appressoria* single or in loose groups, medium brown, irregular in shape, crenate or lobed outline, (10.5–)12(− 23) × (3.5–)5.5(− 11.5) μm. *Sexual morph* on *PDA*. *Ascomata* perithecia, formed after 2 wk., solitary, semi-immersed or immersed in the agar medium, nonstromatic, subspherical to ovoid, ostiolate, glabrous, medium brown, 96–130 × 160–200 μm. *Peridium* 10–12.5 μm thick, composed of pale to medium brown flattened angular cells 3.5–10 μm diam. *Ascogenous hyphae* hyaline, smooth-walled, delicate, rarely visible. *Interascal tissue* not observed. *Asci* unitunicate, 8-spored, cylindrical, tapering to apex and base, smooth-walled, 51–65 × 9.5–13 μm, the base truncate. *Ascospores* biseriately arranged, aseptate, hyaline, smooth-walled, fusiform, slightly curved, base rounded, apex acute or rounded, (13.5–)15–18(− 22) × 5–6(− 6.5) μm,

*Notes*: The conidial length of the isolates examined (CPC 28638, CPC 28639) was variable and fell within the range of the ex-type isolate (CBS 125474) of *C. plurivorum* (15–17 × 5.5 μm; Damm et al. 2019).

*Material examined*: **Thailand**: Chiang Rai, from leaf lesions of *Capsicum annuum*, 7 Jun. 2015, *P.W.J. Taylor* (cultures CPC 28638 and CPC 28639); Bangkok, restaurant in Phaya Thai area, infected tissue of *Capsicum* sp. Jun. 2015, *P.W.J. Taylor* (culture UOM 1004).

### *Colletotrichum scovillei* Damm et al., *Stud. Mycol*. 73: 100 (2012).

*Decription*: *Colonies* on PDA 20–38 mm diam after 7 d (5–6.2 mm/d), flat with entire margin; surface covered with short light pink to orange aerial mycelium, turn grey with time, margin whitish to pale pink, reverse rosy buff, olivaceous grey to brown-grey in the centre; reverse orange to salmon, dark at the centre. *Chlamydospores* not observed. *Conidiomata* acervular, setae not observed, *Conidiophores* hyaline, septate, branched. *Conidiogenous cells* hyaline, cylindrical or ampulliform, 7–17.5 μm, apex 1–3 μm diam. *Conidia* hyaline, aseptate, smooth-walled, mostely fusiform, one end rounded, one end acute, (5.5–)9.5–10(− 12) × (2.5–)3(− 4). Conidia in mass with salmon to orange colour. *Appressoria* single or in loose groups, medium brown, ovoid, entire to crenate margin, (4–)5.5(− 12.5) × (3.5–)4.5–5(− 6.5) μm.

*Notes*: The majority of isolates identified as *C. scovillei* had similar spore shape and spore sizes, compared to the type specimen (10.5–)12.5–15(− 16.5) × (3–)3.5–4(− 4.5) μm, described by Damm et al. (2012a, 2012b). However, some isolates had varying colony colour, different colony growth rates and small differences in spore measurements.

*Material examined*: **Indonesia**: Gowa, from fruit lesions of *Capsicum annuum*, 7 Jun. 2015, *P.W.J. Taylor & A. Nasruddin* (cultures CPC 28577 and CPC 28579); West Java: from fruit lesion of *Capsicum annuum*, Dec. 2014, *F. Fitriyah*, UOM 1146/ EWINDO 10. **Thailand**: Chiang Mai: from fruit lesions of *Capsicum* sp*.* 2008*, O. Mongkolporn* (cultures UOM 1101/313, UOM 1111).

### *Colletotrichum siamense* Prihast. et al., *Fung. Diversity* 39: 98 (2009)

*Description*: *Colonies* on PDA 79 mm diam in 7 d (5.5–6 mm/d). Pale yellow-white, grey, dense cottony aerial mycelium with orange acervular conidiomata at the centre; reverse pale yellowish. *Chlamydospores* not observed. *Conidiomata* acervular, conidiophores formed on a cushion of roundish and medium brown cells. *Setae* not observed. *Conidiophores* hyaline, branched. *Conidiogenous cells* hyaline, cylindrical to ampulliform, 6.5–16 μm. *Conidia* hyaline, aseptate, smooth-walled, fusiform to cylindrical, both ends bluntly rounded, (13–)14(− 15.5) × (3–)4(− 5.5) μm. *Appressoria* dark brown, solitary, circular, entire to crenate margin, (4.5–)7.5(− 10) × (3.5–)3(− 5.5) μm.

*Notes*: *Colletotrichum siamense* isolates from different countries showed variation of morphological characters, in growth rates and culture morphology on PDA. Representative conidial measurements for isolates representing different subclades in the phylogenetic trees (Figs. 2, 3) are: CPC 30233 (Gowa, Indonesia), 12.5–17 × 2.5–5.5 μm; UOM 1132 (Ratchaburi, Thailand) 9.5–14.5 × 3.5–5 μm; UOM 1126/ F4-1C (Kanchana Buri, Thailand) 12–15 × 5–7 μm; UOM 1116 (Kandy, Sri Lanka) 10.5–16.5 × 3.5–5.5. These morphological characters within a subclade were highly consistent within each country. The species was described by Prihastuti et al. (2009); conidia of the ex-holotype specimen (ICMP 18578/ BDP-I2) were reported as 7–18.3 × 3–4.3 μm (*x* = 10.18 ± 1.74 × 3.46 ± 0.36), which encompasses the range observed in our isolates. This species was reported to be biologically and geographically diverse, and is found on many hosts across several tropical and subtropical regions (Weir et al. 2012).

*Material examined*: **Indonesia**: Gowa, from fruit lesion of *Capsicum annuum*, 7 Jun. 2015, *P.W.J. Taylor & A. Nasruddin* (culture CPC 30233); Jeneponto, from fruit lesion of *Capsicum* sp. 7 Jun. 2015, *P.W.J. Taylor & A. Nasruddin* (culture CPC 30209). **Thailand**: Ratchaburi, from fruit lesion of *Capsicum* sp., Jan. 2010, *P.W.J. Taylor & O. Mongkolporn* (culture UOM 1132); Kanchanaburi, from fruit lesion of *Capsicum* sp. Jan. 2010, *P.W.J. Taylor & O. Mongkolporn* (culture UOM 1126 = F4-1C). **Sri Lanka**: Kandy, from fruit lesion of *Capsicum* sp. Sep. 2013, *D.D. De Silva & N. Ranathunge* (culture UOM 1116).

### *Colletotrichum tropicale* E.I. Rojas et al., *Mycologia* 102: 1331 (2010)

*Description*: *Colonies* on PDA 45 mm diam in 7 d (6.5 mm/d). Colonies flat, spreading, with moderate aerial mycelium, On OA surface smoke grey. On PDA surface olivaceous grey to smoke grey, reverse olivaceous grey, numerous orange conidiomata scattered over the surface. On MEA surface dirty white, reverse ochreous. *Asexual morph* on OA. *Vegetative mycelium* consisting of hyaline to pale brown, smooth-walled, septate, branched, 2–2.5 μm diam hyphae. *Chlamydospores* not observed. *Setae* rare (only two seen), straight, medium brown, finely verruculose, 2–3-septate, to 120 μm long, apex subobtusely rounded. *Conidiomata* acervular, 150–250 μm diam, with orange conidial mass. *Conidiophores* subcylindrical, flexuous, 1–3-septate, hyaline, smooth, branched, 15–25 × 3.5–4.5 μm. *Conidiogenous cells* subcylindrical, hyaline, smooth, phialidic with periclinal thickening, 7–15 × 3.5–4.5 μm. *Conidia* hyaline, smooth, aseptate, subcylindrical, straight, apex obtuse, tapering at base to protruding truncate hilum, 1.5–2 μm diam, prominently guttulate, (13–)14–16(− 17) × (3.5–)4–5(− 6) μm. *Appressoria* not observed*. Sexual morph* not observed.

*Material examined*: **Indonesia**, Makassar, from fruit lesion of *Capsicum annuum*, 7 Jun. 2015, *P.W.J. Taylor & A. Nasruddin* (culture CPC 28607).

### Prevalence of sampled *Colletotrichum* species

**Fig. 9:**
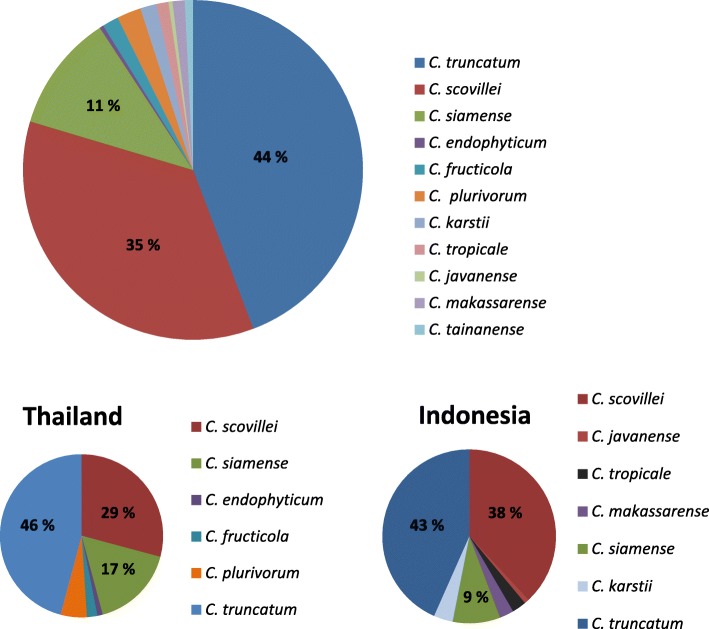
Prevalence of *Colletotrichum* species as a percentage of the total isolates collected in all regions and in Indonesia and Thailand specifically

Overall, *C. truncatum* was the most prevalent species (44%) isolated from infected chili fruit (Fig. 9) and was readily identified by its falcate spores and abundant setae in the necrotic lesions. Of the species with straight conidia, *C. scovillei* (acutatum complex), was the most common species throughout the surveyed countries (35%), except for Sri Lanka where this species was not isolated. *Colletotrichum siamense* (gloeosporioides complex) was the next most common species that occurred in Thailand, Sri Lanka and Indonesia (11%). The remaining species were represented by fewer than 10% of the total number of isolates.

In Indonesia, *C. scovillei* was isolated from infected chili fruit in all surveyed regencies of South Sulawesi, except in Makassar, and in the field trial site in West Java. *Colletotrichum siamense* was also isolated from throughout the region, from Gowa, Jeneponto and Makassar. The two new species, *C. makassarense* and *C. javanense* were isolated from Makassar and West Java, respectively.

In Thailand, *C. scovillei* was isolated mostly from the northern provinces of Chiang Mai and Chiang Rai, but was also obtained from infected chili fruit in a trial site of Kasetsart University in Nakhon Pathom. The Western provinces had a high incidence of *C. siamense* and one isolate of *C. endophyticum* was collected from Kanchana Buri. *Colletotrichum plurivorum* was isolated from chili leaves with necrotic lesions in Chiang Rai and from necrotic lesions of chili fruit found in a restaurant in Phaya Thai area of Bangkok. In addition, *C. fructicola* was identified from fruit collected from Chiang Mai.

In Taiwan, an isolate was identified as a new species, *C. tainanense,* collected from infected chili fruit in the Tainan province, and *C. fructicola* was identified from fruit in Hsinchu and Nantou, Taiwan. *Colletotrichum plurivorum* was also identified from a fruit collected in Johor, Malaysia.

### Pathogenicity

All the *Colletotrichum* species caused anthracnose symptoms on wounded fruit, but there were significant differences in severity of the symptoms. On wounded fruit, isolates of *C. scovillei* and *C. javanense* showed the highest disease severity, producing large, necrotic lesions with mean lesion sizes 15.6–20.3 mm (Table 5). On non-wounded fruits, all species were less pathogenic, with mean lesion sizes less than 10 mm. *Colletotrichum makassarense, C. tropicale* and *C. plurivorum,* produced only very small lesions or no visible symptoms (mean lesion size < 1 mm) 10 d after inoculation of non-wounded fruits. *Colletotrichum javanense* and *C. scovillei* isolates were the most pathogenic in non-wounded fruit and produced lesions with mean sizes of 9.4 and 9.1 mm respectively. Lesions caused by *C. scovillei* were significantly larger than those caused by all other species except for *C. javanense* in wounded fruit and *C. endophyticum* in unwounded fruit. Significance of the pairwise t-tests is strongly dependent on the number of isolates of each species sampled, so the groupings shown in Table 5 do not simply change with the magnitude of the difference of the means; a large difference may not be significant if there are small sample sizes for both species while a smaller difference may be significant. In particular, comparisons involving *C. javanense* and *C. endophyticum* are very imprecise as only one isolate of each species was tested.

**Table 5:** Mean lesion size of symptoms caused by *Colletotrichum* species with straight conidia on inoculated mature red fruit of *Capsicum annuum* cv. Bangchang

Wound inoculation				Non-wound inoculation			
Species	Least squares mean mm	Standard Error	t-grouping^a^	Species	Least squares mean mm	Standard Error	t-grouping^a^
*C. scovillei*	20.3	0.09	a	*C. javanense*	9.4	0.28	ab
*C. javanense*	15.6	0.35	ab	*C. scovillei*	9.1	0.07	a
*C. siamense*	9.7	0.11	bc	*C. fructicola*	3.6	0.2	bc
*C. karsti*	9.4	0.2	bcd	*C. endophyticum*	2.8	0.29	bc
*C. fructicola*	7.8	0.25	be	*C. karsti*	2	0.16	c
*C. tainanense*	6.9	0.25	be	*C. tainanense*	1.5	0.2	c
*C. makassarense*	6.4	0.18	ce	*C. siamense*	1.3	0.09	c
*C. plurivorum*	5.3	0.16	de	*C. makassarense*	< 1.0	0.14	c
*C. endophyticum*	4.4	0.35	ce	*C. tropicale*	< 1.0	0.16	c
*C. tropicale*	4.1	0.2	de	*C. plurivorum*	< 1.0	0.13	c
control	0	0.35	e	control	0	0.29	c

^a^Pairwise t-tests between species least square means, significant differences at alpha = 0.05 level are indicated by different letters

## DISCUSSION

*Colletotrichum* isolates collected from infected fruit and leaf tissue of chili plants from Thailand, Indonesia, Taiwan, Sri Lanka, and Malaysia were allocated to different species complexes with 11 *Colletotrichum* species being identified and three new species described.

*Colletotrichum truncatum* was the most prevalent species of *Colletotrichum* causing anthracnose of chili in Asia, which supports previous reports of *C. truncatum* being widely distributed on chili fruit throughout Asia, Australia, and South America (Sharma et al. 2014; De Silva et al. 2017a; Diao et al. 2017; Mongkolporn and Taylor 2018). *Colletotrichum truncatum* has a broad host range infecting many crop species (https://nt.ars-grin.gov/fungaldatabases/; Ranathunge and Hewa Bajjamage 2016).

The remaining 56% of isolates with straight conidia belonged to the gloeosporioides, acutatum, boninense and orchidearum species complexes. Of these, *C. scovillei*, in the acutatum complex, was the most prominent species (35% of the total isolates) and was distributed in Indonesia, Malaysia, Thailand and Taiwan. However, *C. scovillei* was not identified in the survey of Sri Lanka, possibly because of the small number of samples assessed. Previous studies have also reported the predominance of *C. scovillei* in Asia and Brazil (Diao et al. 2017, Mongkolporn and Taylor 2018), but this species has not been identified in Australia (De Silva et al. 2017a) and hence remains an important biosecurity threat to the Australian capsicum/chili industry. Further surveys are required to confirm the presence of *C. scovillei* in Australia and Sri Lanka.

*Colletotrichum siamense*, in the gloeosporioides complex, was also prominent (11% of total isolates) in causing anthracnose of chili fruit in Indonesia, Sri Lanka and Thailand, but was not detected in Malaysia and Taiwan. Nevertheless, Noor and Zakaria (2018) reported the identification of *C. siamense* in Malaysia possibly due to a larger sampling size of infected chili across states in Malaysia. Within the *C. siamense* clade, small subclades were observed that coincided with different geographical regions from where the isolates were collected*. Colletotrichum siamense* has been reported to infect chili in Asia, Australia, Brazil, and is a common pathogen of many other plant species (Weir et al. 2012; James et al. 2014; Sharma and Shenoy 2014; Liu et al. 2016a, 2016b; de Oliveira et al. 2017; De Silva et al. 2017a; Diao et al. 2017; Suwannarat et al. 2017).

*Colletotrichum siamense* isolates from different countries appeared to show different morphological characters with varying growth rates and culture morphologies. This variability in morphological characters indicated that this taxon has high intra-specific diversity. The combined gene analyses of *ApMat* and *gs* sequences also supported the distinction of subclades within the *C. siamense* clade. In the last few years, there has been significant debate on whether *C. siamense s. lat.* should be separated into different species groups within the gloeosporioides complex, with the number of accepted species ranging from one to seven (Weir et al. 2012; Udayanga et al. 2013). However, a recent case study concluded that *C. siamense s. lat.* was a single species rather than a species complex as no independent evolutionary lineages were found within this species (Liu et al. 2016a).

*Colletotrichum tropicale* is reported for the first time as causing anthracnose in *Capsicum* in Indonesia*.* Rojas et al. (2010) noted that *C. tropicale* was initially isolated from a wide range of hosts in forests in tropical America, from rotting fruit and as a leaf endophyte. Silva et al. (2017) recently reported *C. tropicale* causing chili anthracnose in Brazil. *Colletotrichum tropicale* was also reported from Japan, Panama, Thailand, and from other host species (Mongkolporn and Taylor 2018).

*Colletotrichum fructicola* is reported for the first time causing chili anthracnose in Thailand and Taiwan. *Colletotrichum fructicola* was previously reported to cause anthracnose in chili from India and China (Sharma and Shenoy 2014; Diao et al. 2017). Prihastuti et al. (2009) originally isolated *C. fructicola* from coffee berries in Thailand, and then *C. fructicola* was reported as a leaf endophyte from several plants in South America (Weir et al. 2012; Vieira et al. 2014) *Colletotrichum fructicola* has a wide host range and was reported by Weir et al. (2012) as a biologically and geographically diverse species.

All the species in the gloeosporioides complex were identified using combined multi-locus gene analyses, based on the ITS*, gapdh, chs-1, act* and *tub2* genes, which showed higher diversity on chili than those in the acutatum, boninense and orchidearum species complexes. Phylogenetic tree provided good resolution of the species with high support values, which supported the species boundaries and identified the novel species.

Phylogenetic trees built from *ApMat* and *gs* gene sequences had similar topologies to the multigene phylogenetic tree confirming that the *ApMat* and *gs* loci were highly informative and that they distinguished most species in the gloeosporioides species complex (Silva et al. 2012; Sharma et al. 2013; Liu et al. 2015). However, some recent new species (Diao et al. 2017; Marin-Felix et al. 2017) were unable to be placed in the *ApMat* and *gs* phylogenetic tree due to the absence of the *ApMat* gene sequence data. Although there were reports that the *gs* gene alone is not a good marker for differentiating *C. siamense* isolates (Weir et al. 2012), these data showed multiple base pair differences in gene sequences of the *gs* loci of *C. siamense* species similar to the *ApMat* locus. In addition, the lack of noticeable subclading in *C. siamense in the 5-gene tree* compared to the *ApMat* and *gs* tree*,* confirmed that the *ApMat* and gs loci were more informative than the other five gene loci. The *ApMat* gene has been shown previously to improve the systematics of the gloeosporioides species complex, providing complementary phylogenetic information compared to other loci (Silva et al. 2012). Liu et al. (2015) also applied the *ApMAT* gene in a more recent molecular phylogenetic analyses of the species in this complex and discussed the merit of using *ApMat* and *ApMat* in combination with *gs* to resolve the phylogeny.

Although four isolates from Indonesia were identified as *C. karsti* in the boninense complex, they formed a subclade within the *C. karsti* species clade and had different conidial sizes to the ex-type strain of *C. karsti* (Yang et al. 2011), suggesting that these might be a new species. However, sufficient phylogenetic support was not observed in all the individual gene trees to justify the introduction of a novel species. Besides, Damm et al. (2012a, 2012b) reported that the conidium size of *C. karsti* was quite variable. *Colletotrichum karsti* has been reported from China and India to cause anthracnose disease in *Capsicum* spp. (Liu et al. 2016b; Saini et al. 2016; Diao et al. 2017). *Colletotrichum karsti* has the widest known host range and distribution of all species in the boninense complex (Damm et al. 2012b). Most of the *C. karsti* strains had been isolated as endophytes but a few were derived from diseased plant tissues. This species has mostly been isolated from dicotyledonous plants, but some have occurred on monocotyledonous families, especially *Orchidaceae* and *Musaceae* (Damm et al. 2012b).

*Colletotrichum plurivorum* was identified for the first time causing anthracnose in Thailand and Malaysia. The five *C. plurivorum* isolates from chili in Thailand formed a distinct subclade with high support values within the *C. plurivorum* subclade, and separated from *C. cliviicola* (syn. *C. cliviae*, Damm et al. 2019)*.* In addition, the *C. plurivorum* isolates formed a characteristic sexual morph in culture, which was not reported for *C. cliviicola* (Yang et al. 2009*)*.

Recently, Damm et al. (2019) resolved the taxonomic placement of several *Colletotrichum* strains which did not belong to any of the accepted species complexes and assigned them to three new species complexes including the orchidearum complex. Recent studies in China and Brazil also identified multiple species belonging to these complexes, including *C. brevisporum, C. cliviicola*, *C. liaoningense,* and *C. plurivorum* that caused anthracnose disease in chili (Liu et al. 2016b; De Silva et al. 2017b; Diao et al. 2017). *Colletotrichum plurivorum* belongs to the orchidearum complex with many isolates reported to have a large host range (Damm et al. 2019). The type specimen was described as new from *Coffea* in Vietnam (Nguyen et al. 2010). *Colletotrichum plurivorum* was originally described as *C. sichuanensis* from *Capsicum annuum* in the Sichuan Province of China (Liu et al. 2016b). However, the name was invalid, because no holotype specimen was cited (Mongkolporn and Taylor 2018; Damm et al. 2019).

Pathogenicity tests of *Colletotrichum* spp. from chili showed that while all the species were pathogenic on chili fruits after wounding the fruit surface, most produced a low level of infection on non-wounded fruit. This illustrates the importance of the cuticle acting as a barrier to infection by *Colletotrichum* spp. (Auyong et al. 2015) and emphasises the need for informed and standardised inoculation techniques in pathogenicity assays. Some species such as *C. tropicale, C. makassarense* and *C. plurivorum* which produced a low level of infection in the assays on non-wounded fruit, may have a predominantly endophytic lifestyle then switch to a necrotrophic life style to complete their life-cycle (De Silva et al. 2017b). However, further pathogenicity tests on different chili cultivars and at different fruit maturity stages are necessary to comprehensively evaluate their pathogenicity. Pathogenicity testing of *C. plurivorum* on chili leaves and fruits showed that the isolates collected from Chiang Rai and Malaysia could infect leaves but not fruit (results not shown) and suggested they might be specialised leaf pathogens. In contrast, two isolates of *C. plurivorum* from Bangkok did not infect leaves but did infect wounded fruits. These results demonstrate the pathogenic variation that can exist within a single species.

Mongkolporn et al. (2010) identified pathotypes of *C. truncatum*, *C. scovillei* (as *C. acutatum*) and *C. siamense* (as *C. gloeosporioides*) within isolates of each species from Thailand. Pathotypes were identified by inoculating wounded fruit of *Capsicum baccatum* and *C. chinense* genotypes. All the isolates identified as *C. gloeosporioides* and *C. acutatum* in Mongkolporn et al. (2010) were subsequently re-identified as *C. siamense* and *C. scovillei*, respectively except for isolate UOM 1137 (F5-2D), which was identified as *C. endophyticum*. The isolate UOM 1137 was pathogenic in both the wound and non-wound bioassays, and was classified in the most virulent *C. siamense* pathotype group (PCg1-R) in Mongkolporn et al. (2010). This contrasts with the study by Manamgoda et al. (2013) where *C. endophyticum* was described as an endophyte of *Pennisetum purpureum*. The severity of infection in chili may indicate that *Capsicum annuum* was the preferred host for *C. endophyticum* and *P. purpureum* was a less favoured host, where the pathogen infected but existed in an endophytic lifestyle. In addition, isolate UOM 1137 also has shorter spores than the type isolate of *C. endophyticum,* thus further isolates of this species need to be collected from chili plants and *P. purpureum* in Thailand to confirm taxonomy and pathogenicity.

## CONCLUSIONS

Multigene phylogenetic analyses of *Colletotrichum* species causing anthracnose disease of *Capsicum* in Asia showed high species diversity with the identification of 11 different *Colletotrichum* species, including three novel species. Although *C. siamense* has been reported as infecting many plant species before, this was the first report of *C. siamense* causing anthracnose in chili in Indonesia and Sri Lanka. This was also the first report of *C. fructicola* infecting chili in Thailand and Taiwan. In addition, all three novel species were new additions to the *Colletotrichum* species causing anthracnose in chili. More surveys in countries in Asia and Oceania need to be conducted to identify the diversity and prevalence of species causing chili anthracnose. Understanding of the taxonomy and the pathogenicity of *Colletotrichum* spp. has great significance to fruit and vegetable industries, where there are serious biosecurity implications of incursion by exotic pathogens.

## Abbreviations

AGRF: Australian Genome Research Facility
BI: Bayesian inference analyses
CBS: Westerdijk Fungal Biodiversity Institute, The Netherlands
CI: Consistency index
DIC: Differential interference contrast
DNA: Deoxyribonucleic acid
MCMC: Markov chain Monte Carlo algorithm
MEA: Malt extract agar
ML: Maximum likelihood
MP: Maximum parsimony
OA: Oatmeal agar
PAUP: Phylogenetic analysis using parsimony
PCR: Polymerase chain reaction
PDA: Potato dextrose agar
PP: Posterior probabilities
RC: Rescaled consistency index
RI: Retention index
SNA: Synthetic nutrient-poor agar
TBR: Tree bisection and reconstruction
TL: Tree length
UOM: University of Melbourne
WA: Water agar
